# FGL2‐HDAC11 Drives Immunothrombosis via NETs‐Mediated Endothelial Capillarization in MASLD Fibrosis

**DOI:** 10.1002/advs.202522985

**Published:** 2026-05-10

**Authors:** Xitang Li, Junjian Hu, Suping Hai, Peng Hu, Wenhui Wu, Qiang Gao, Binghui Yu, Feiyang Xu, Xizhe Zheng, Qianting Guan, Huiling Xiang, Dong Xi, Weiming Yan, Peng Wang, Bin‐hao Zhang, Qin Ning, Xiaojing Wang

**Affiliations:** ^1^ Department of Infectious Diseases Tongji Medical College and State Key Laboratory for Diagnosis and Treatment of Severe Zoonotic Infectious Diseases, Tongji Hospital Huazhong University of Science and Technology Wuhan Hubei China; ^2^ Hepatic Surgery Center, Institute of Hepato‐Pancreato‐Biliary Surgery, Department of Surgery, Tongji Hospital, Tongji Medical College Huazhong University of Science and Technology Wuhan Hubei China

**Keywords:** fibrinogen like protein 2, immunothrombosis, liver fibrosis, metabolic dysfunction‐associated steatotic liver disease, neutrophil extracellular traps

## Abstract

Metabolic dysfunction–associated steatotic liver disease (MASLD) is frequently accompanied by hepatic fibrosis and systemic cardiovascular complications; however, the mechanistic interplay between coagulation abnormalities and disease progression remains poorly defined. Here, analyses of liver tissues and plasma from patients with MASLD, together with complementary mouse models, suggest an important role of immunothrombosis in fibrotic progression. In MASLD mouse models, pharmacological anticoagulation with dabigatran or aspirin attenuates fibrosis but increases systemic bleeding risk, highlighting the need for more selective strategies. Mechanistically, neutrophil extracellular traps (NETs) promote localized fibrin deposition within the hepatic microvasculature, leading to impaired microcirculation and liver sinusoidal endothelial cell (LSEC) capillarization associated with increased Piezo1‐dependent mechanosensation, thereby exacerbating fibrosis. Further investigation identifies neutrophil‐derived fibrinogen‐like protein 2 (FGL2) as a key upstream regulator of NETs formation through interaction with histone deacetylase 11 (HDAC11), promoting histone H3 deacetylation and facilitating PAD4‐mediated citrullination to drive NETs release. Genetic disruption of FGL2 or NETs inhibition restores LSEC fenestration, improves microvascular hemodynamics, and attenuates fibrosis without increasing systemic bleeding risk. Together, these findings define an immunothrombotic axis linking neutrophil‐derived FGL2–HDAC11 signaling to NETs formation and endothelial dysfunction in MASLD, providing mechanistic insight into the interplay between coagulation and metabolic liver disease.

## Graphical abstract

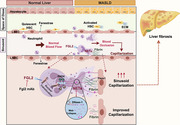

**Abbreviations**
ALT: alanine aminotransferase
AST: aspartate aminotransferase
FGL2: fibrinogen‐like protein 2
HDAC11: histone deacetylase 11
HFD: high‐fat diet; LSEC, liver sinusoidal endothelial cell
MASLD: metabolic dysfunction‐associated steatotic liver disease
MCD: methionine‐choline‐deficient
MPO: myeloperoxidase
NAS: NAFLD Active Score
NE: neutrophil elastase
NETs: neutrophil extracellular traps
PA: Palmitic Acid

## 1 Introduction

Metabolic dysfunction‐associated steatotic liver disease (MASLD) is the most prevalent chronic liver disorder globally, affecting approximately 38% of the population and significantly elevating risks of liver fibrosis and hepatocellular carcinoma [1, 2]. The recent redefinition of MASLD emphasizes its inherent cardiometabolic implications [3], multiple studies have demonstrated that MASLD is associated with a 1.33‐fold increased odds of coronary heart disease and a 1.45‐fold higher hazard of cardiovascular events, independent of metabolic syndrome [4, 5, 6]. This suggests that hepatic steatosis may drive vascular dysfunction, a key yet incompletely characterized aspect of MASLD pathogenesis.

**SCHEME 1:**
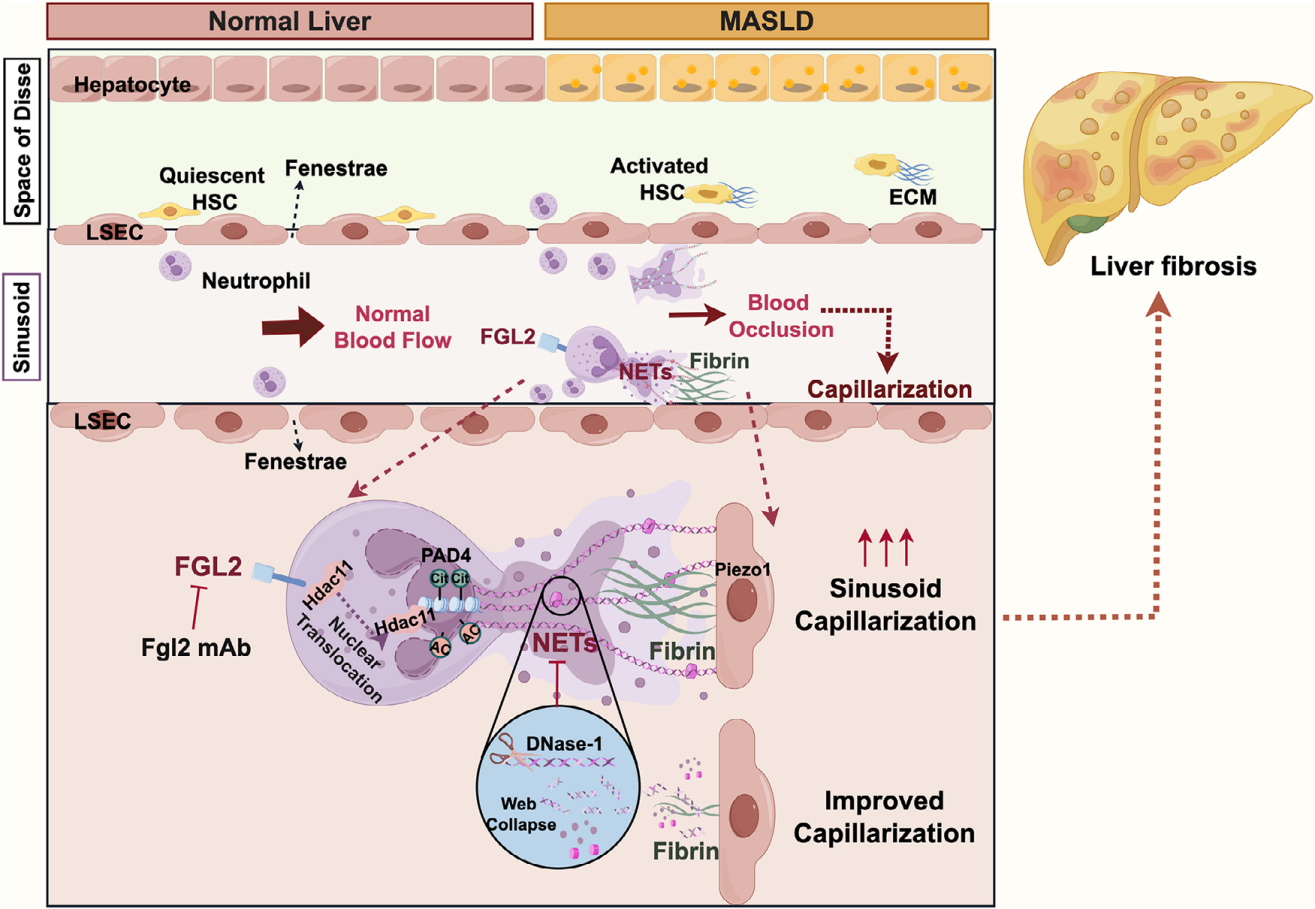
Schematic representation of the role of FGL2‐mediated immunothrombosis in liver fibrosis progression in MASLD. In the normal liver, liver sinusoidal endothelial cells (LSEC) maintain fenestration and normal blood flow, supported by quiescent hepatic stellate cells (HSC). In contrast, in MASLD, FGL2+ neutrophils are recruited to the liver, where they release neutrophil extracellular traps (NETs), leading to fibrin deposition, blood occlusion, and LSEC capillarization. Mechanistically, FGL2 binds to HDAC11, promoting histone deacetylation and PAD4‐mediated citrullination, which drives NETs formation. This process is associated with increased Piezo1 expression, which exacerbates fibrosis. Inhibition of FGL2 using an FGL2‐specific monoclonal antibody (mAb) or DNase‐1 treatment prevents NET formation, restores LSEC fenestration, improves microvascular hemodynamics, and attenuates fibrosis. These findings establish a mechanistic link between immunothrombosis and liver fibrosis in MASLD.

While obesity‐related vascular injury has been extensively investigated [7], liver‐specific coagulopathy in MASLD remains poorly elucidated. Notably, despite normal standard coagulation parameters (e.g., prothrombin time, activated partial thromboplastin time), patients with MASLD exhibit upregulated coagulation factors VIII and IX, accompanied by intrahepatic fibrin deposition that correlates with the severity of liver fibrosis [8, 9, 10]. A recent real‐world study further revealed that treatment with the antiplatelet agent aspirin significantly alleviates inflammation and fibrosis in MASLD [11], suggesting that targeting the coagulation system may offer a novel therapeutic approach with currently limited treatment options [12]. However, the risk of systemic bleeding limits the clinical application of traditional anticoagulants [13, 14], underscoring the need to explore liver‐specific mechanisms of coagulopathy to develop safer intervention strategies.

The concept of immunothrombosis, defined as coagulation orchestrated by innate immune cells, provides a framework for tissue‐specific coagulopathy [15]. Unlike systemic anticoagulation, targeting the “immune” component may mitigate hypercoagulability without compromising hemostasis. Among key effectors of immunothrombosis, neutrophil extracellular traps (NETs) occupy a central role [16]. These web‐like structures consist of chromatin filaments decorated with cytotoxic proteases and histones [17, 18], which substantially expand the functional surface area of neutrophils [19]. Recent studies have shown that NETs contribute to deep vein thrombosis by entrapping platelets, fibrinogen, and other coagulation factors to establish a prothrombotic niche [20]. Although the unique microcirculatory architecture of the liver, characterized by relative slow blood flow and abundant blood supply, facilitates the formation and retention of NETs [19], few studies have explored the relationship between NETs release and coagulation activation in MASLD‐affected liver tissues. Additionally, previous studies have reported elevated levels of neutrophil‐attracting chemokines in MASLD, including CXCL1/CXCL2, S100A8/A9, and LCN2, which promote hepatic neutrophil recruitment [21, 22, 23]. Moreover, in vitro studies have confirmed that free fatty acids could induce NETs formation [24]. Thus, evaluating the role and mechanism of NETs‐mediated coagulation in MASLD‐related fibrosis is critical for elucidating hepatic immunothrombosis and identifying safer intervention strategies.

Building on our previous findings, we have shown that fibrinogen‐like protein 2 (FGL2), a pivotal molecule with both prothrombinase activity and immunomodulatory functions [25], drives NETs formation in fulminant hepatitis [26]. However, the regulatory role and precise mechanisms of FGL2 in NETs formation and coagulopathy within the context of MASLD remain unclear. A hallmark of MASLD is neutrophil infiltration within hepatic sinusoids, where immune cells establish close contact with liver sinusoidal endothelial cells (LSECs) [27]. LSECs possess unique fenestrated structures that confer high permeability, a feature critical for maintaining hepatic metabolic homeostasis [28]. During MASLD progression, LSECs undergo capillarization, characterized by fenestrae loss and basement membrane, a process that promotes hepatic steatosis and fibrosis [28, 29]. Nevertheless, the underlying mechanisms driving this pathological transformation remain elusive, and the potential involvement of NETs in this process has not been established.

In this study, we demonstrate that FGL2 interacts with histone deacetylase 11 (HDAC11) to regulate the acetylation‐citrullination balance of histone H3, thereby inducing NETs formation. These NETs subsequently activate local immunothrombotic cascades, promoting microthrombus formation and LSEC capillarization. Importantly, targeted inhibition of either FGL2 or NETs effectively ameliorates sinusoidal microcirculatory dysfunction without compromising systemic hemostatic function. Collectively, these findings suggest a potential role for the FGL2–NETs–LSEC axis in MASLD‐associated fibrogenesis and indicate that this pathway may represent a potential therapeutic target. (Scheme 1).

## 2 Materials and Methods

### 2.1 Human Subjects

Liver tissues samples and plasma specimens were collected from 14 patients diagnosed with MASLD and 10 control subjects. The diagnosis of MASLD was made in accordance with international clinical guidelines, with secondary causes of hepatic steatosis (e.g., alcoholic liver disease, drug‐induced steatosis) excluded [30, 31]. The research was conducted in compliance with the Declarations of Helsinki and Istanbul. All participants gave written informed consent. All experimental protocols involving human samples were reviewed and approved by the Clinical Trial Ethics Committee of Huazhong University of Science and Technology (Approval No.: 2024S091)

### 2.2 Animal Models

To establish MASLD mouse models with concurrent fibrosis, C57BL/6 wild‐type (WT) mice and fgl2 knockout (*fgl2^−/−^)* mice were assigned to two dietary intervention groups, including a high‐fat diet (HFD) for 36 weeks or a methionine‐choline deficient diet (MCD) for 5 weeks. For NETs depletion, mice were administered DNase‐1 via intraperitoneal injection at a dose of 2.5 mg/kg, with injections performed three times per week. In neutrophil adoptive transfer experiments, recipient mice received 4 × 10^6^ neutrophils per week for 3 consecutive weeks to assess the role of neutrophils in MASLD pathogenesis. All experimental protocols were reviewed and approved by the Animal Ethics Committee of Tongji Hospital (Approval No.: TJH‐202311018). Detailed procedures for materials preparation and experimental operations are provided in the Supplementary Materials and Methods.

### 2.3 Statistical Analysis

All experimental data were expressed as the mean ± standard error of the mean (SEM). For statistical comparisons, two‐group comparisons were performed using an unpaired Student's *t*‐test, multiple‐group comparisons were analyzed via one‐way analysis of variance (one‐way ANOVA), followed by Tukey's post hoc test for pairwise comparisons to correct for multiple testing. Statistical significance was defined as a two‐tailed *p*‐value < 0.05 (*p* < 0.05). All statistical analyses were conducted using GraphPad Prism software.

## 3 Results

### 3.1 Coagulation Activation and Microcirculatory Impairment in MASLD

**FIGURE 1:**
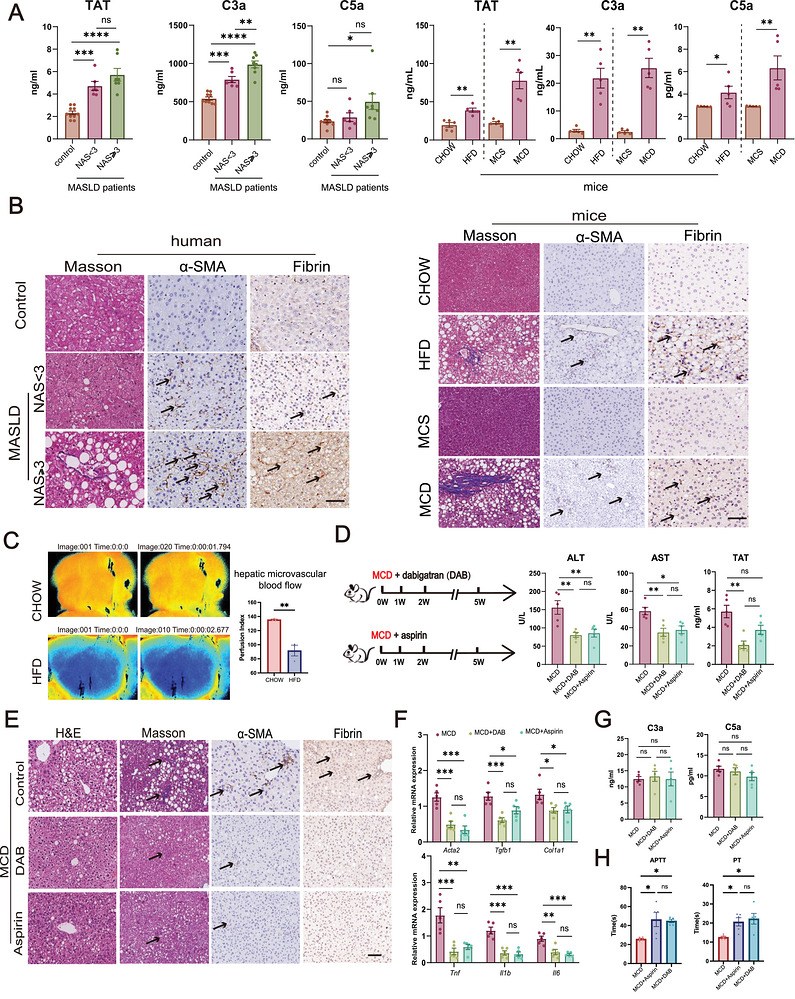
Coagulation Dysregulation During MASLD Progression and Therapeutic Efficacy of Coagulation Pathway Inhibition.(A) Plasma concentrations of key coagulation (TAT) and complement (C3a,C5a) components, in patients with MASLD (*n* = 14) and mouse models (*n* = 5 in each group), compared with their respective control groups.(B) Representative hepatic tissue sections showing Masson's trichrome staining (collagen deposition), α‐smooth muscle actin (α‐SMA) immunohistochemistry (hepatic stellate cell activation), and fibrin deposition in patients with MASLD, animal models, and control subjects (*n* = 3 in each group).(C) Real‐time quantification of hepatic microvascular blood flow velocity via laser speckle contrast imaging (LSCI) in chow‐fed control mice and HFD‐induced MASLD mice (*n* = 3 in each group).(D) Schematic of the experimental design for anticoagulant treatment (aspirin or dabigatran) in MCD diet‐induced MASLD mice. Therapeutic effects on liver enzymes (ALT, AST) and TAT levels post‐anticoagulation (*n* = 5 in each group).(E) Histopathological evaluation (H&E, Masson's trichrome, α‐SMA, fibrin deposition) post‐therapeutic intervention (*n* = 5 in each group). (F) Hepatic mRNA expression of inflammatory cytokine profiles (*Inf‐α, Il‐1β, Il‐6*) and fibrosis‐related genes (*Acta2, Tgfb1, Col1a1*) after therapeutic intervention with anticoagulants (*n* = 5 in each group). G) Plasma levels of complement components C3a and C5a following anticoagulant treatment in MASLD mice (*n* = 5 in each group). (H) Effects on coagulation parameters (APTT, PT) following anticoagulant administration (*n* = 5 in each group). Scale bar: 50 µm. *p < .05,**p < .01, ***p < .001, and ****p < .0001.

This study enrolled 14 patients with MASLD and 10 control subjects, the detailed clinical characteristics of the participants were summarized in Table S1. We first analyzed the expression profiles of coagulation and complement components, and identified significant upregulation of thrombin‐antithrombin complex (TAT), complement component 3a (C3a), and complement component 5a (C5a) in both patients with MASLD and experimental mouse models (Figure 1A). Concurrently, histopathological analysis revealed enhanced hepatic collagen accumulation and fibrin deposition in these subjects (Figure 1B). Notably, fibrin deposition reflects the formation of stable fibrin networks via thrombin‐mediated fibrinogen conversion, a process that is central to thrombosis initiation and progression [32].

To dynamically evaluate hepatic microcirculation, we employed laser speckle contrast imaging (LSCI), a technique enabling real‐time in vivo monitoring of microvascular perfusion [33]. Quantitative analysis showed that the microcirculatory flow velocity was reduced by approximately 50% in HFD mice compared with control mice (Figure 1C), providing direct in vivo evidence of coagulation dysfunction coupled with microcirculatory impairment in MASLD.

To systematically assess the therapeutic potential of targeting coagulation pathways, we administered the antiplatelet agent aspirin or the direct thrombin inhibitor dabigatran to MCD diet fed mice. Both interventions effectively mitigated systemic hypercoagulability, as indicated by reduced TAT levels (Figure 1D), attenuated intrahepatic microthrombosis, accompanied by significant reductions in hepatic inflammation and fibrogenesis (Figure 1E,F). However, neither treatment modulated the degree of complement activation (Figure 1G). Importantly, both therapeutic strategies significantly prolonged the bleeding time in treated mice (Figure 1H), a critical clinical limitation of conventional coagulation‐targeting drugs, as increased bleeding risk restricts their application.

We also evaluated the effects of these interventions on hepatic metabolic parameters. Neither aspirin nor dabigatran altered liver‐to‐body weight ratios (Figure S1A). Further analysis showed that dabigatran partially improved hepatic lipid metabolism, whereas aspirin exerted minimal effects on lipid homeostasis (Figure S1B).

Collectively, our findings highlight the importance of identifying tissue‐specific coagulation targets that can be precisely modulated to alleviate MASLD‐related pathology while preserving systemic hemostatic function, thereby addressing the limitations of existing anticoagulant strategies and paving the way for safer and more effective therapeutic interventions.

### 3.2 NETs Drive Immunothrombosis and Exacerbate the Progression of MASLD

**FIGURE 2:**
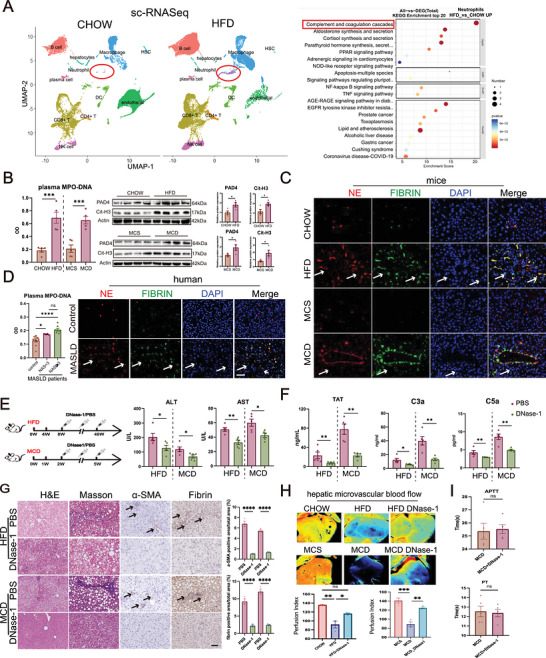
Enhanced NETs contribute to coagulopathy and exacerbates MASLD progression. (A) Comparative analysis of neutrophil infiltration in HFD‐fed versus chow‐fed mice via single‐cell RNA sequencing; Kyoto Encyclopedia of Genes and Genomes (KEGG) pathway enrichment of differentially expressed genes in hepatic neutrophils from HFD mice compared with chow mice (*n* = 3 in each group).(B) Quantification of plasma myeloperoxidase‐DNA (MPO‐DNA) and hepatic expression of NETs markers (citrullinated histone H3, Cit‐H3; peptidyl arginine deiminase 4, PAD4) levels quantified in MASLD mice (*n* = 5 in each group). (C,D) Immunofluorescence co‐localization of NETs (neutrophil elastase, NE, red) and fibrin (green) deposits in murine (C) and human (D) livers (*n* = 3 in each group); plasma MPO‐DNA was also detected in human MASLD samples. (E) Experimental timeline for DNase‐1 intervention in MASLD mice; plasma levels of liver enzymes (ALT, AST) levels post‐treatment (*n* = 5 in each group). (F) Therapeutic effects of DNase‐1 treatment on coagulation (TAT) and complement (C3a, C5a) activation markers (*n* = 5 in each group). (G) Histopathological evaluation (H&E, Masson's trichrome, α‐SMA) and detection of fibrin deposition post‐DNase‐1treatment (*n* = 3 in each group). (H) Assessment of hepatic microcirculatory improvement via laser speckle imaging post‐treatment (*n* = 3 in each group). (I) Systemic coagulation parameters (APTT, TT) after DNase‐1 administration (*n* = 5 in each group). Scale bar: 50 µm, *p < .05, **p < .01, ***p < .001, and ****p < .0001.

Next, we analyzed single‐cell RNA sequencing (scRNA‐seq) data of hepatic non‐parenchymal cells isolated from chow‐fed control mice and HFD‐fed mice (36 weeks of feeding). Results showed a significant increase in neutrophil number in steatotic livers. Functional enrichment analysis further demonstrated that these neutrophils were enriched in pathways related to complement and coagulation system activation (Figure 2A), suggesting their active involvement in coagulation processes. Previous research has shown that neutrophils primarily facilitate coagulation activation via the formation of NETs [16]. Consistent with this, we examined NETs expression in both murine and human MASLD liver tissue and observed markedly elevated NETs levels (Figure 2B–D). Notably, we also found extensive colocalization between NETs and fibrin deposits, a key structural component of thrombi (Figure 2C,D).

We administered deoxyribonuclease I (DNase‐1) to degrade NETs in both HFD‐ and MCD‐fed mice (Figure 2E), and found this intervention led to a significant decrease in hepatic NETs content (Figure S2A). Concurrently, we observed reduced plasma levels of liver enzymes (ALT, AST; Figure 2E) and decreased circulating concentrations of TAT, C3a, and C5a (Figure 2F). DNase‐1 treatment also alleviated hepatic histopathological damage, reduced inflammation and liver fibrosis (Figure 2G; Figure S2B,C), and improved hepatic microcirculation (Figure 2H), with minimal effects on APTT and PT (Figure 2I). Furthermore, NETs depletion normalized lipid metabolic parameters (Figure S2E). These findings demonstrate that NETs‐mediated thrombus formation contributes to hepatic microcirculatory dysfunction, exacerbates MASLD‐associated inflammation and fibrosis, and does not disrupt systemic hemostatic balance.

### 3.3 Neutrophil‐Derived FGL2 Mediates NETs Formation and Coagulopathy in MASLD

**FIGURE 3:**
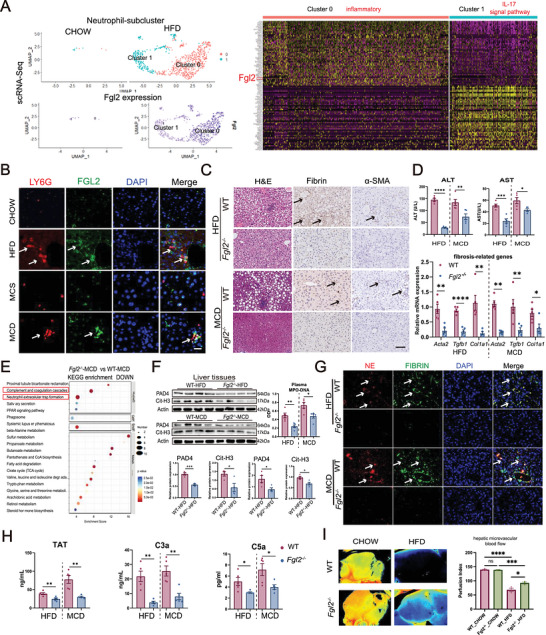
FGL2 exacerbates MASLD progression by mediating NETs release and subsequent coagulation dysregulation.(A) ScRNA‐seq analysis of neutrophil subpopulations in HFD‐fed versus chow‐fed mice. Expression profiles of marker genes in hepatic neutrophil clusters and FGL2 expression patterns across neutrophil subsets.(B) Immunofluorescence detection of FGL2 in hepatic neutrophils (LY6G, red; FGL2, green) from MASLD mice (*n* = 3 in each group).(C) Histopathological evaluation (H&E, Masson's trichrome, α‐SMA, fibrin) in WT and *fgl2^−/−^* mice (*n* = 3 in each group).(D) Plasma ALT and AST levels in *fgl2^−/−^* mice under HFD and MCD dietary challenges. Hepatic fibrogenic markers (*Acta2, Tgfb1, Col1a1*) expression in WT and *fgl2^−/−^* mice (*n* = 5 in each group). (E) KEGG pathway analysis of differentially expressed genes in *fgl2^−/−^* and WT mice fed an MCD diet. F) Western blot analysis of NETs markers (PAD4, Cit‐H3) in hepatic tissues. Plasma levels of MPO‐DNA in WT and *fgl2^−/−^* mice (*n* = 5 in each group).(G) Immunofluorescence co‐staining of NETs (NE, red) and fibrin (green) in liver sections (*n* = 3 in each group).(H) Coagulation (TAT) and complement (C3a, C5a) activation in WT and *fgl2^−/−^* mice (*n* = 5 in each group).(I) Assessment of hepatic microcirculatory improvement via laser speckle imaging in WT and *fgl2^−/−^* mice (*n* = 3 in each group). Scale bar = 50 µm. *p < .05, **p < .01, ***p < .001, and ****p < .0001.

Subpopulation analysis of hepatic neutrophils using scRNA‐seq data revealed two distinct clusters (Cluster 0 and Cluster 1), with Cluster 0 exhibiting significantly higher expression of FGL2 (Figure 3A) and functional enrichment in inflammatory responses (Figure S3A). While our previous work in fulminant hepatitis demonstrated FGL2‐mediated NETs release [34], the regulatory role of FGL2 in MASLD remained unclear. Immunofluorescence and sc‐RNA seq analysis confirmed elevated FGL2 expression in hepatic neutrophils from MASLD mice (Figure 3B, Figure S3B).

Next, we generated *fgl2^−/−^* mice and established both MCD and HFD models of MASLD. Genetic ablation of FGL2 significantly ameliorated hepatic inflammation and fibrosis in both dietary models (Figure 3C,D). Consistently, *Cxcl1* and *Cxcl2* expression was significantly reduced in *fgl2^−/−^* mice, indicative of impaired chemokine‐mediated neutrophil recruitment (Figure S3C). Transcriptomic profiling revealed coordinated downregulation of two critical pathways in *fgl2^−/−^* mice: complement and coagulation cascade activation and neutrophil extracellular trap formation (Figure 3E; Figure S3D).

Consistent with these findings, *fgl2^−/−^* mice exhibited a marked reduction in hepatic and plasma NETs markers under both dietary regimens (Figure 3F; Figure S3E), accompanied by alleviated hepatic fibrin deposition (Figure 3G) and decreased levels of coagulation activation products (TAT) and complement activation fragments (C3a, C5a) (Figure 3H). Notably, laser speckle contrast imaging revealed enhanced hepatic microcirculation in *fgl2^−/−^* mice, demonstrating increased blood flow velocity (Figure 3I). Furthermore, *fgl2* deficiency partially improved lipid metabolic profiles (Figure S3F). Taken together, these data established that FGL2 expressed by neutrophils promoted NETs release, thereby inducing coagulation dysfunction and fibrin accumulation, which collectively drive liver inflammation and accelerate fibrogenesis.

**FIGURE 4:**
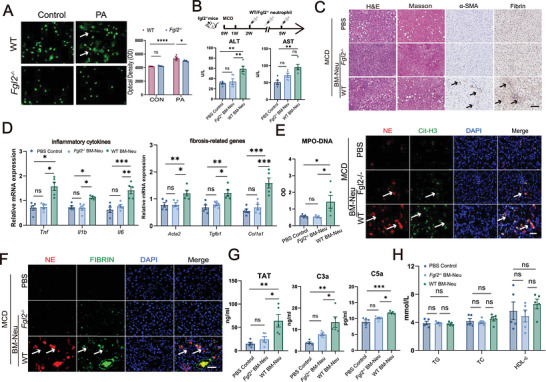
Neutrophil‐derived FGL2 exacerbates MASLD progression by mediating NETs formation and subsequent coagulopathy.(A) NETs release assessment via SYTOX Green staining and fluorometric quantification in PA‐stimulated bone marrow neutrophils from WT and *fgl2^−/−^* mice (*n* = 5 in each group).(B) Experimental schema for adoptive transfer of WT or *fgl2^−/−^* bone marrow‐derived neutrophils into *fgl2^−/−^* recipients fed an MCD diet. Plasma aminotransferase (ALT, AST) levels in recipients (*n* = 5 in each group).(C) Histopathological evaluation (H&E, Masson's trichrome, α‐SMA, fibrin) in recipients (*n* = 3 in each group).(D) Hepatic expression of inflammatory cytokines (*Tnf‐α, Il‐1β, Il‐6*) and fibrogenic genes (*Acta2, Tgfb1, Col1a1*) in recipients (*n* = 5 in each group).(E) Plasma MPO‐DNA complexes in recipients (*n* = 5 in each group). Hepatic NETs formation post‐transfer (NE, red; Cit‐H3, green) (*n* = 3 in each group).(F) Immunofluorescence co‐localization of NETs (NE, red) and fibrin (green) in liver sections (*n* = 3 in each group).(G) Coagulation (TAT) and complement (C3a, C5a) activation profiles in recipients (*n* = 5 in each group).(H) Plasma lipid metabolic profiles in recipients (*n* = 5 in each group). Scale bar = 50 µm. *p < .05, **p < .01, ***p < .001, and ****p < .0001.

While our study initially utilized global fgl2 knockout mice, this approach cannot definitively establish the neutrophil‐derived functions of FGL2. We conducted additional experiments to verify this mechanism. First, we isolated neutrophils from WT and *fgl2^−/−^* mice and stimulated them with palmitic acid (PA) in vitro. Neutrophils lacking FGL2 demonstrated significantly impaired NETs release capacity compared to WT controls (Figure 4A). To further validate these findings in vivo, we performed neutrophil adoptive transfer experiments. We transplanted either WT or *fgl2^−/−^* bone marrow‐derived neutrophils into *fgl2^−/−^* recipient mice fed an MCD diet. Notably, recipient mice that received *fgl2^−/−^* neutrophils exhibited significantly attenuated liver injury (Figure 4B) and ameliorated hepatic histopathological damage and fibrosis, findings supported by both histological staining and fibrogenic gene expression analysis (Figure 4C,D).

Recipients of *fgl2^−/−^* neutrophils also showed reduced hepatic and plasma NETs levels compared to those receiving WT neutrophils (Figure 4E), accompanied by significantly diminished fibrin deposition on NETs in liver tissues (Figure 4F). Consistent with the reduction in NETs formation, recipient mice of *fgl2^−^/^−^* neutrophils exhibited decreased plasma levels of coagulation (TAT) and complement (C3a, C5a) activation markers (Figure 4G). However, neutrophil‐derived FGL2 deficiency did not significantly affect lipid metabolism parameters (Figure 4H). These results demonstrate that neutrophil‐derived FGL2 plays a critical role in MASLD pathogenesis by specifically regulating NETs formation, thereby promoting coagulopathy, fibrin deposition, and subsequent hepatic inflammation and fibrosis.

### 3.4 FGL2 Regulates NETs Formation Through an HDAC11‐Associated Epigenetic Mechanism

**FIGURE 5:**
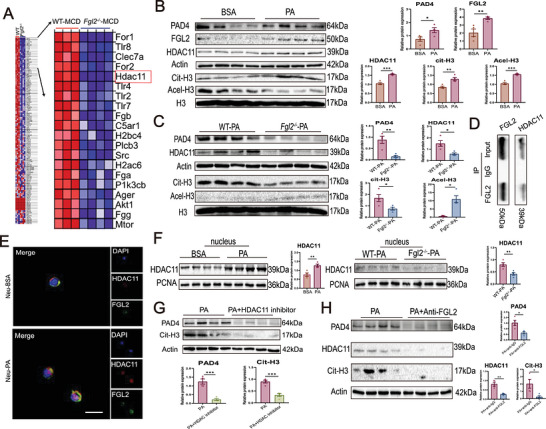
Neutrophil‐derived FGL2 promotes NETs release via HDAC11 regulation. (A) Heatmap analysis of NETs‐related gene expression profiles from transcriptomic sequencing of nonparenchymal cell isolated from *fgl2^−/−^* mice and WT controls. (B) Western blot analysis of HDAC11, FGL2, Cit‐H3, and Acel‐H3 in WT neutrophils after PA‐stimulated. (C) Western blot comparison of HDAC11, FGL2, Cit‐H3, and Ac‐H3 expression differences in neutrophils from WT mice and *fgl2^−/−^* mice after PA stimulation. (D) Co‐immunoprecipitation between FGL2 and HDAC11. (E) Immunofluorescence co‐localization of FGL2 and HDAC11 in PA‐activated WT neutrophils. (F) Nuclear HDAC11 expression levels in neutrophils from WT mice and *fgl2^−/−^* mice after PA treatment. (G) Effect of HDAC11 inhibitor (elevenostat, 1 µM) on PAD4 and Cit‐H3 expression in PA‐stimulated WT neutrophils. (H) Impact of FGL2‐neutralizing antibody on HDAC11 activity and expression levels of PAD4 and Cit‐H3. Scale bar = 50 µm; *n* = 3–5 in each group, *p < .05, **p < .01, ***p < .001, and ****p < .0001.

Although this study has confirmed the regulatory role of neutrophil‐derived FGL2 in the formation of NETs, its underlying molecular mechanisms remain incompletely elucidated. Transcriptomic analysis of NETs‐related genes revealed significant downregulation of histone deacetylase 11 (*Hdac11*) in *fgl2^−/−^* mice (Figure 5A). As the sole member of class IV histone deacetylases (HDACs), HDAC11 exhibits low sequence homology with other HDAC family members and primarily mediates histone deacetylation [35]. However, its potential role in NETs formation has not been reported.

Therefore, this study further investigated the regulatory effect of FGL2 on HDAC11 and the impact of HDAC11 on NETs release. First, in vitro experiment showed that stimulation with PA significantly upregulated the deacetylase HDAC11 in WT neutrophils, accompanied by a decrease in histone acetylation level (Acel‐H3) and an increase in the expression levels of PAD4 and Cit‐H3, key regulatory factors of NETs formation (Figure 5B). After PA stimulation, *fgl2^−/−^* neutrophils exhibited reduced HDAC11 expression, along with increased Acel‐H3 level and significantly decreased expression levels of PAD4 and Cit‐H3 levels (Figure 5C). Co‐immunoprecipitation assay results demonstrated a direct interaction between FGL2 and HDAC11 (Figure 5D). Immunofluorescence colocalization analysis confirmed the colocalization and revealed that upregulated HDAC11 nuclear translocation following stimulation (Figure 5E), a critical step for its deacetylase activity [35]. Western blot analysis of neutrophil nuclear extracts further confirmed the nuclear accumulation of HDAC11 following PA treatment. In contrast, HDAC11 levels were significantly reduced in the nuclei of neutrophils from *fgl2^−/−^* mice (Figure 5F).

To clarify the functional role of HDAC11, an HDAC11‐specific inhibitor [36, 37], was used for intervention in this study. The results showed that this inhibitor effectively suppressed NETs release (Figure 5G). In addition, a recombinant antibody targeting the N‐terminal procoagulant domain of FGL2 was constructed. In vitro blocking experiments confirmed that this antibody could inhibit HDAC11‐mediated histone deacetylation and thereby suppress NETs formation (Figure 5H). Collectively, these findings indicate that neutrophil‐derived FGL2 promotes NETs release through an HDAC11‐associated epigenetic mechanism, thereby exacerbating coagulation dysfunction and driving the progression of hepatic inflammation and fibrosis in MASLD. Moreover, the precise mechanisms regulatory mechanism linking coagulation disorders to liver fibrosis in MASLD remains unclear and requires further in‐depth investigation.

### 3.5 NETs‐Induced Microthrombosis is Associated With Endothelial Capillarization and Piezo1 Upregulation

**FIGURE 6:**
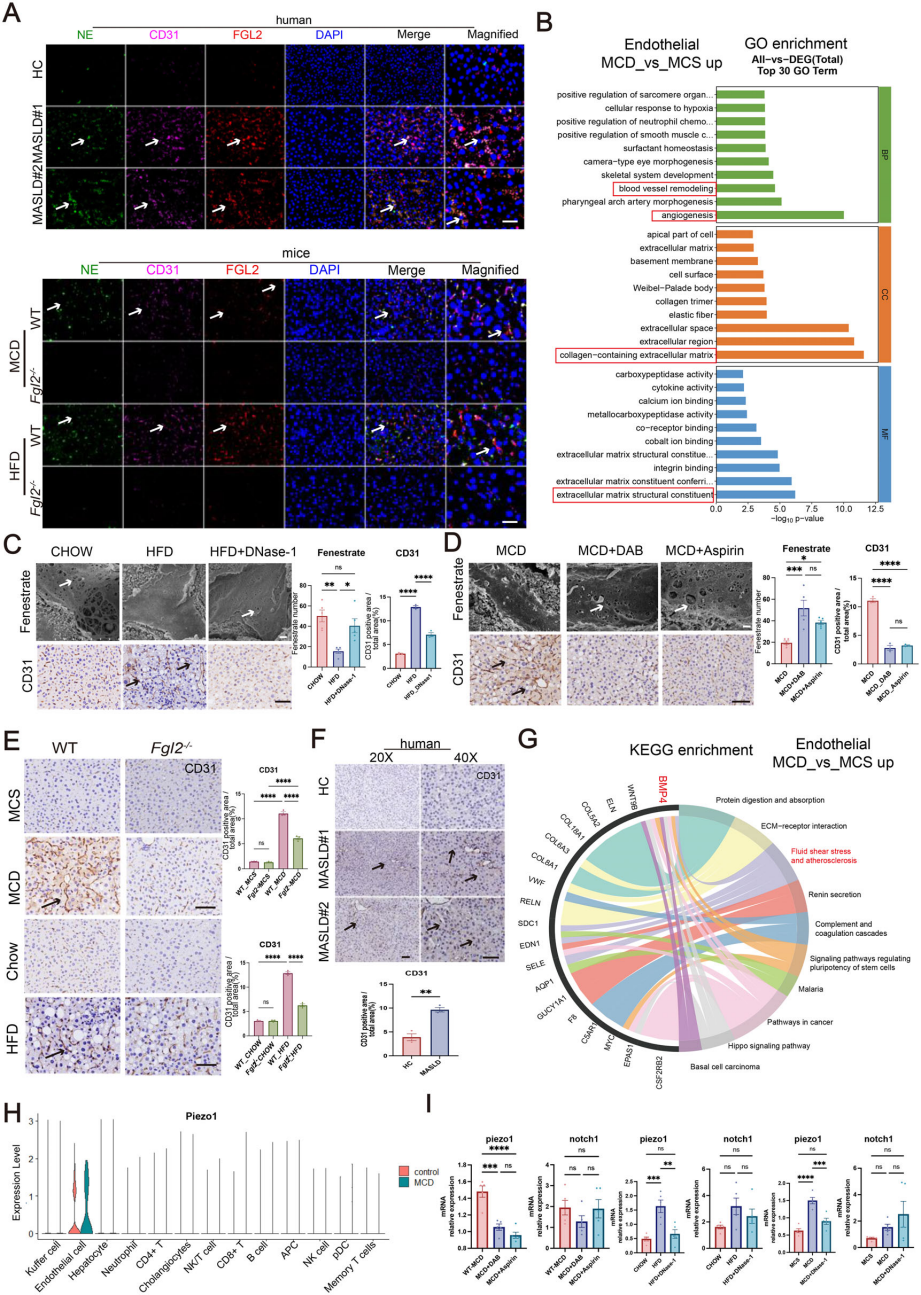
NETs promote microthrombosis and drive MASLD progression via Piezo1‐associated endothelial capillarization. (A) Triple immunofluorescence of NETs (NE; green), endothelial cells (CD31; magenta), and FGL2 (red) in liver tissues of human and mice (Scale bar = 50 µm). (B) GO pathway enrichment analysis of genes significantly upregulated in endothelial cells from MCD‐fed mice compared with those from WT mice. (C,D) Scanning electron microscopy (SEM) of hepatic sinusoid fenestrations (Scale bar = 0.2 µm) and CD31 staining (Scale bar = 50 µm) in mice treated with DNase‐1 (C) and anticoagulants (D). (E) Perisinusoidal CD31 distribution in WT and *fgl2^−/−^* mice, scale bar = 50 µm. (F) Perisinusoidal CD31 distribution in patients with MASLD (Scale bar = 50 µm). (G) Chord diagram showing key genes and pathways enriched in KEGG analysis of significantly upregulated genes in endothelial cells from MCD‐fed mice compared with those from WT mice. (H) Violin plot from single‐cell sequencing showing Piezo1 expression across different cell subpopulations in both WT and MCD‐fed mice. (I) Expression levels of Piezo1 and Notch1 in endothelial cells following anticoagulation or NETs depletion. *n* = 3–5 in each group, *p < .05, **p < .01, ***p < .001, and ****p < .0001.

Spatially, we observed colocalization of FGL2, NETs, and fibrin along the hepatic sinusoid microvasculature in patients with MASLD (Figure S4A), indicating that FGL2‐mediated NETs formation may contribute to sinusoidal endothelial cell injury, which requires further investigation. Multiplex staining of liver tissues also confirmed the colocalization of NETs and the endothelial cell marker CD31 (Figure 6A). Subsequently, we integrated scRNA‐Seq data of non‐parenchymal cells from MCD‐fed mice, after quality control, marker gene identification, and cell type annotation (Figure S4B–D), we extracted endothelial cells and analyzed the functional enrichment of genes significantly upregulated in endothelial cells from MCD‐fed mice compared with those from WT mice. These upregulated genes were significantly enriched in biological processes related to blood vessel remodeling and angiogenesis (Figure 6B), suggesting that LSECs might exhibit impaired function in MASLD.

Given the extensive colocalization of NETs and LSECs, we hypothesize that NETs may induce endothelial cell injury, particularly endothelial defenestration and capillarization, thereby contributing to the progression of MASLD. Endothelial defenestration refers to the loss of fenestrations, structures unique to endothelial cells lacking a basement membrane. This loss impairs nutrient delivery and metabolic exchange between hepatocytes and circulating blood, which are essential for maintaining proper hepatic metabolic function [28]. Endothelial capillarization is accompanied by the formation of new capillaries. This process is supported by evidence of upregulated perisinusoidal CD31 expression [38]. This capillarization process abolishes the LSECs' normal capacity to quiescent hepatic stellate cells (HSCs), leading to HSC activation and subsequent fibrogenesis [29, 39, 40]. Importantly, capillarization abolishes the ability of LSECs to keep hepatic stellate cells (HSCs) in a quiescent state, leading to HSC activation and subsequent fibrogenesis

Therefore, we first investigated the effect of NETs on endothelial defenestration and capillarization. We found that NETs depletion improved LSEC fenestration and reduced capillarization (Figure 6C; Figure S4E), confirming that NETs exacerbate endothelial dysfunction and hepatic fibrosis. Furthermore, we evaluated the role of coagulation in endothelial injury and capillarization. Our results showed that anticoagulant treatment partially restored fenestration density and reduced CD31 staining (Figure 6D). Similarly, FGL2 is involved in this process, as endothelial capillarization was significantly ameliorated in fgl2^−^/^−^ mice (Figure 6E). Concurrently, endothelial capillarization was also observed in patients with MASLD (Figure 6F).

Further KEGG pathway enrichment analysis revealed that BMP4 was associated with several pathways, including Fluid shear stress and atherosclerosis, Pathways in cancer, the Hippo signaling pathway and Basel cell in carcinoma (Figure 6G). Given the close relationship between endothelial capillarization and altered intrahepatic hemodynamic forces, we focused on the Fluid shear stress and atherosclerosis pathway, which is closely linked to endothelial mechanotransduction. Among genes involved in this pathway, Piezo1 and Notch1 have been reported as mechanosensitive regulators of endothelial remodeling [41, 42, 43, 44]. Notably, Piezo1 has also been implicated in the regulation of BMP signaling [45, 46], suggesting a potential connection between mechanical stress and BMP4‐related pathways. Analysis of the scRNA‐seq dataset revealed that Piezo1 was predominantly expressed in endothelial cells (Figure 6H), whereas Notch1 did not exhibit endothelial enrichment (Figure S4F). Moreover, Piezo1 expression was significantly increased in MCD‐fed mice (Figure 6H). Importantly, both anticoagulation treatment and NETs depletion markedly reduced Piezo1 expression, while Notch1 expression remained largely unchanged (Figure 6I). These results suggest that restoration of intrahepatic hemodynamics preferentially modulates mechanosensitive signaling pathways involving Piezo1 in endothelial cells.

Collectively, these findings demonstrate that FGL2 promotes NETs release and microthrombus formation, leading to altered shear stress and Piezo1 upregulation, which are associated with endothelial capillarization and the progression of MASLD.

### 3.6 Therapeutic Targeting of FGL2 Alleviates MASLD Without Bleeding Risks

**FIGURE 7:**
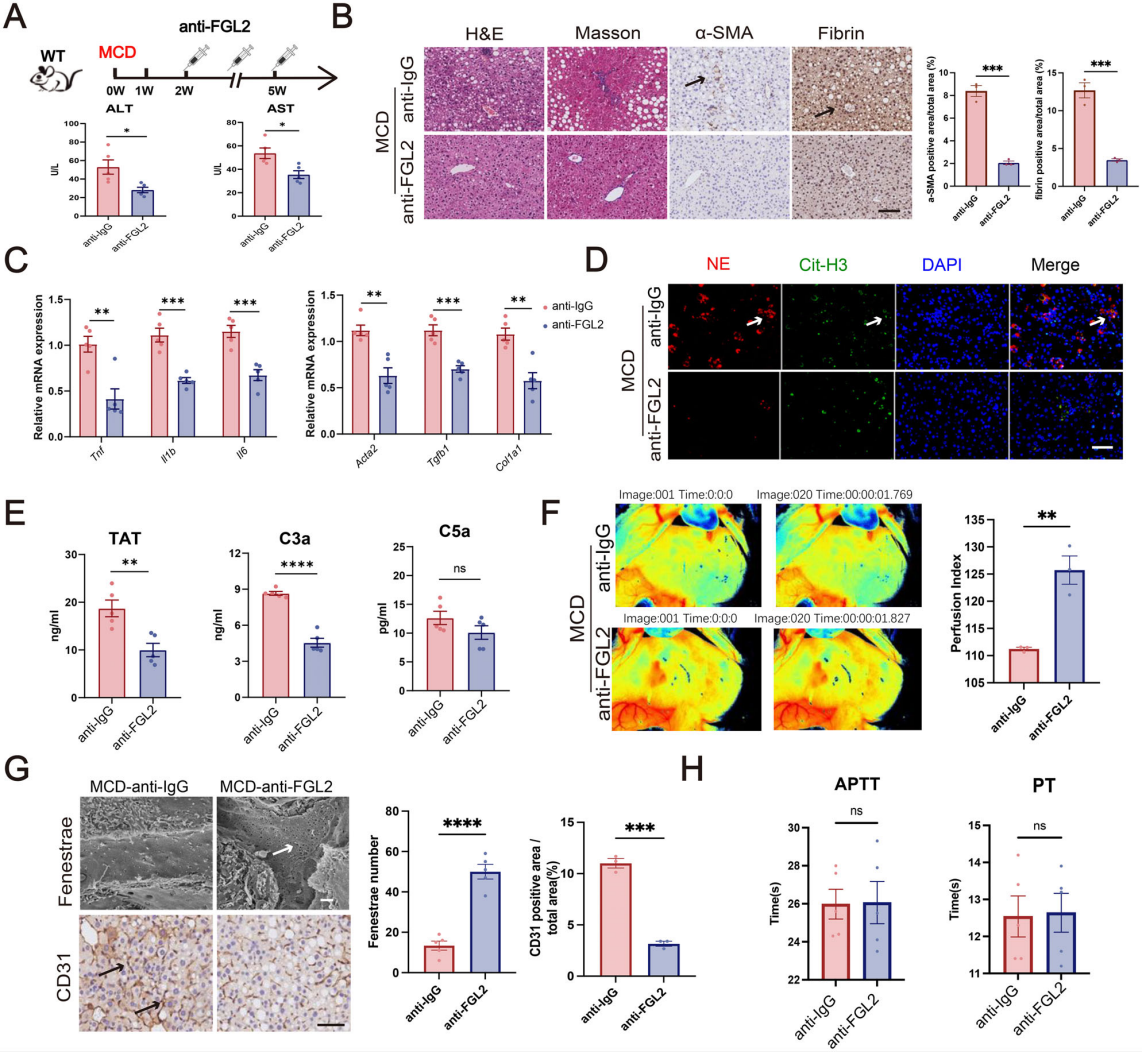
FGL2 monoclonal antibody therapy attenuates NETs and subsequent microthrombosis, ameliorating endothelial capillarization. (A) Schematic of the experimental protocol for FGL2‐neutralizing antibody treatment in MASLD mice. Plasma aminotransferase levels (ALT, AST) as indicators of hepatocellular injury. (B) Histopathological evaluation including H&E staining (hepatocyte injury), Masson's trichrome (collagen deposition), α‐SMA immunohistochemistry (activated stellate cells), and fibrin staining (microthrombosis), scale bar = 50 µm. (C) mRNA expression levels of proinflammatory cytokine profiles (*Tnf‐α, Il‐1β, Il‐6*) and fibrogenic markers (*Acta2, Tgfb1, Col1a1*). (D) Immunofluorescence analysis of hepatic NETs formation (NE, red; Cit‐H3, green) following FGL2 antibody administration, scale bar = 50 µm. (E) Plasma levels of coagulation (TAT) and complement (C3a, C5a) molecules following FGL2 mAb treatment. (F) In vivo assessment of hepatic microcirculatory improvement using laser speckle contrast imaging. (G) Ultrastructural analysis of sinusoidal endothelial fenestration by scanning electron microscopy (scale bar = 0.2 µm) with corresponding CD31 immunohistochemistry (scale bar = 50 µm) demonstrating reversal of capillarization. (H) Systemic coagulation parameters (activated partial thromboplastin time, APTT; thrombin time, TT). *p < .05, **p < .01, ***p < .001, and ****p < .0001.

Subsequently, we developed a recombinant antibody targeting the procoagulant N‐terminal domain of FGL2 and evaluated its therapeutic efficacy in MASLD progression. Treatment with FGL2 monoclonal antibody (mAb) significantly decreased the levels of liver enzymes, including ALT and AST (Figure 7A). Following FGL2 mAb treatment, amelioration of hepatic inflammation and fibrosis was observed (Figure 7B,C). Most importantly, FGL2 mAb treatment significantly reduced NETs formation (Figure 7D; Figure S6A). Along with decreased NETs formation, there was a downregulation of procoagulant markers and an improvement in hepatic microcirculatory perfusion (Figure 7E,F). Additionally, scanning electron microscopy revealed partial restoration of LSEC fenestrations and reduced CD31 expression following FGL2 mAb treatment (Figure 7G), indicating attenuation of endothelial capillarization. No significant change of systemic bleeding risk following treatment (Figure 7H). Furthermore, treatment with fgl2 did not alter the lipid metabolic profile (Figure S6B). These findings demonstrate that N‐terminal‐specific FGL2 mAb therapy alleviates MASLD progression by targeting localized coagulopathy without compromising systemic hemostasis, supporting further investigation of FGL2 as a potential therapeutic target.

## 4 Discussion

Our study systematically elucidates the pivotal role of immunothrombosis in MASLD progression. Emerging evidence indicates that thrombin receptors PAR‐1/2 not only regulate hepatic fibrosis but also modulate glucose homeostasis [47, 48], suggesting multifaceted biological effects of the coagulation system in MASLD. While clinical observations confirm a prothrombotic state in patients with MASLD [8, 9, 10], the hemodynamic alterations and the underlying mechanisms in hepatic microcirculation remain unexplored. Using laser speckle imaging, we demonstrate that hepatic microcirculatory flow velocity is reduced by approximately 50% in MASLD mice, accompanied by significant fibrin deposition. Intervention studies with aspirin and dabigatran confirm that inhibiting either thrombin or platelet function ameliorates hepatic inflammation and fibrosis. These findings provide direct evidence that targeting coagulation pathways can mitigate MASLD progression, yet also highlight a key clinical translation challenge, heightened systemic bleeding risk. For instance, vorapaxar was withdrawn due to bleeding risks [49], and both aspirin and dabigatran prolonged bleeding time in our study, underscoring the urgent need to identify key coagulation nodes that can be safely targeted.

As a critical nexus connecting immunity and coagulation [16, 50], NETs were identified as a key driver of hepatic fibrogenesis in our study. Our results showed that NETs promote fibrin deposition and immunothrombosis through concurrent activation of both complement and coagulation cascades. Notably, while dabigatran and aspirin exerted minimal effects on complement activation, DNase‐1 treatment demonstrated superior efficacy by disrupting both coagulation and complement activation.

We also uncover a spatially distinct pattern of NETs‐fibrin complexes along hepatic sinusoids. This discovery provides topological evidence for how NETs exacerbate local tissue injury through microthrombosis in the hepatic immune microenvironment, complementing previous reports that NETs induce metabolic reprogramming of stellate cells in Disse's space [51]. Moreover, recent investigation into tumor microenvironments have revealed that intravascular NETs can disrupt blood flow and induce tumor necrosis by forming physical barriers [52]. This phenomenon aligns with our observations in hepatic sinusoids, collectively underscoring the universal role of NETs in promoting microcirculatory dysfunction across diverse pathological contexts.

To delineate upstream regulators of NETs, we focused on FGL2, a molecule with prothrombinase activity [25]. Here, we identify a novel epigenetic mechanism. FGL2 promotes NETs formation in MASLD by interacting with HDAC11, which is a class IV histone deacetylase. Specifically, FGL2 facilitates HDAC11 nuclear translocation, and this translocation catalyzes histone H3 deacetylation. The deacetylation creates a permissive state for PAD4‐mediated citrullination and subsequent NETs release. While class I/II HDACs have been implicated in PAD4‐dependent histone citrullination [53], our findings suggest that class IV HDAC11 may also participate in the regulation of NETs formation. HDAC11, a unique member of the HDAC family, has emerging roles in lipid metabolism, glucose tolerance, immune regulation, and energy homeostasis [54]. Building on this context, our work further expands the functional relevance of HDAC11 by elucidating novel crosstalk between the coagulation cascade and epigenetic modulation.

Sinusoidal endothelial capillarization represents a critical step in MASLD fibrogenesis [55]. Our intervention studies demonstrate that both NETs depletion and anticoagulation reverse capillarization, as evidenced by restored fenestration and reduced basement membrane deposition, as demonstrated by confocal and transmission electron microscopy. Mechanistically, we observed upregulation of the mechanosensor Piezo1 in MASLD endothelial cells, and this increase was attenuated by anticoagulant or DNase‐1 treatment. Based on previous research [41, 42], we proposed that NETs promote microthrombus formation, leading to vascular stenosis and reduced blood flow.

Laser speckle imaging confirmed reduced hepatic microvascular blood flow, which was associated with increased Piezo1 expression and endothelial capillarization. Together, these findings suggest a framework linking immunothrombosis to fibrosis via biomechanical reprogramming of endothelial cells. These findings further suggest that Piezo1 may represent a mechanosensitive pathway linking NETs‐driven hemodynamic alterations to BMP4‐associated endothelial remodeling during sinusoidal capillarization. However, the causal role of Piezo1 in this process remains to be established, and future investigations should also consider the potential contribution of other factors, such as inflammatory signaling, hypoxia, and extracellular matrix remodeling [55], to LSEC capillarization.

Several limitations warrant consideration. First, although our study demonstrates a dominant role of neutrophil‐derived FGL2 in MASLD‐associated NETs formation and microthrombosis, FGL2 expressed in other cell types may also contribute to disease progression, highlighting the need for future studies using cell‐type–specific knockout models. Moreover, therapeutic interventions were evaluated only in short‐term preclinical models, and the human cohort was observational with limited sample size. Therefore, long‐term safety, including potential risks of infection and immune dysfunction, as well as clinical applicability require validation in future well‐powered studies.

Taken together, our study delineates a previously unrecognized immunothrombotic axis linking neutrophil‐derived FGL2–HDAC11–dependent epigenetic regulation to NETs formation and subsequent endothelial capillarization in MASLD. By integrating metabolic stress, innate immune remodeling, and microvascular dysfunction into a unified framework, these findings refine the current understanding of disease progression and provide a conceptual basis for targeting immunothrombosis in metabolic liver disease.

## Author Contributions

Q.N., X.W., and X.L. designed the project. X.L. and S.H. performed the experiments, acquired and analyzed data with the assistance of P.H. and J.H. J.H. and S.H. provided key interactive information and discussion. W.W., Q.G., B.Y., F.X., Q.G., H.X., D.X., W.Y., and P.W. participated in the data discussion and provided suggestions. B.‐H.Z. provided clinical samples. X.L. wrote the manuscript, Q.N. and X.W. helped revise the manuscript.

## Funding

This work was supported by the National Key Research and Development Program of China (2023YFC2308600), the National Natural Science Foundation of China (No. 82370609, No.82170596), and the National Youth Talent Support Program (No. 0106540082).

## Conflicts of Interest

The authors declare no conflicts of interest.

## Supporting information




**Supporting File 1**: advs75659‐sup‐0001‐SuppMat.docx.

**Supporting File 2**: advs75659‐sup‐0002‐Figure1.pdf.

**Supporting File 3**: advs75659‐sup‐0003‐Figure2.pdf.

**Supporting File 4**: advs75659‐sup‐0004‐Figure3.pdf.

**Supporting File 5**: advs75659‐sup‐0005‐Figure4.pdf.

**Supporting File 6**: advs75659‐sup‐0006‐Figure5.pdf.

## Data Availability

The data that support the findings of this study are available on request from the corresponding author. The data are not publicly available due to privacy or ethical restrictions.
