# Supplementary material for: FGL2‐HDAC11 Drives Immunothrombosis via NETs‐Mediated Endothelial Capillarization in MASLD Fibrosis
Source: Adv Sci (Weinh). 2026 May 10;13(44):e22985. doi: 10.1002/advs.202522985 (PMC13335973; doi:10.1002/advs.202522985)
Supplement: Supplementary file 1 [file ADVS-13-e22985-s001.pdf]

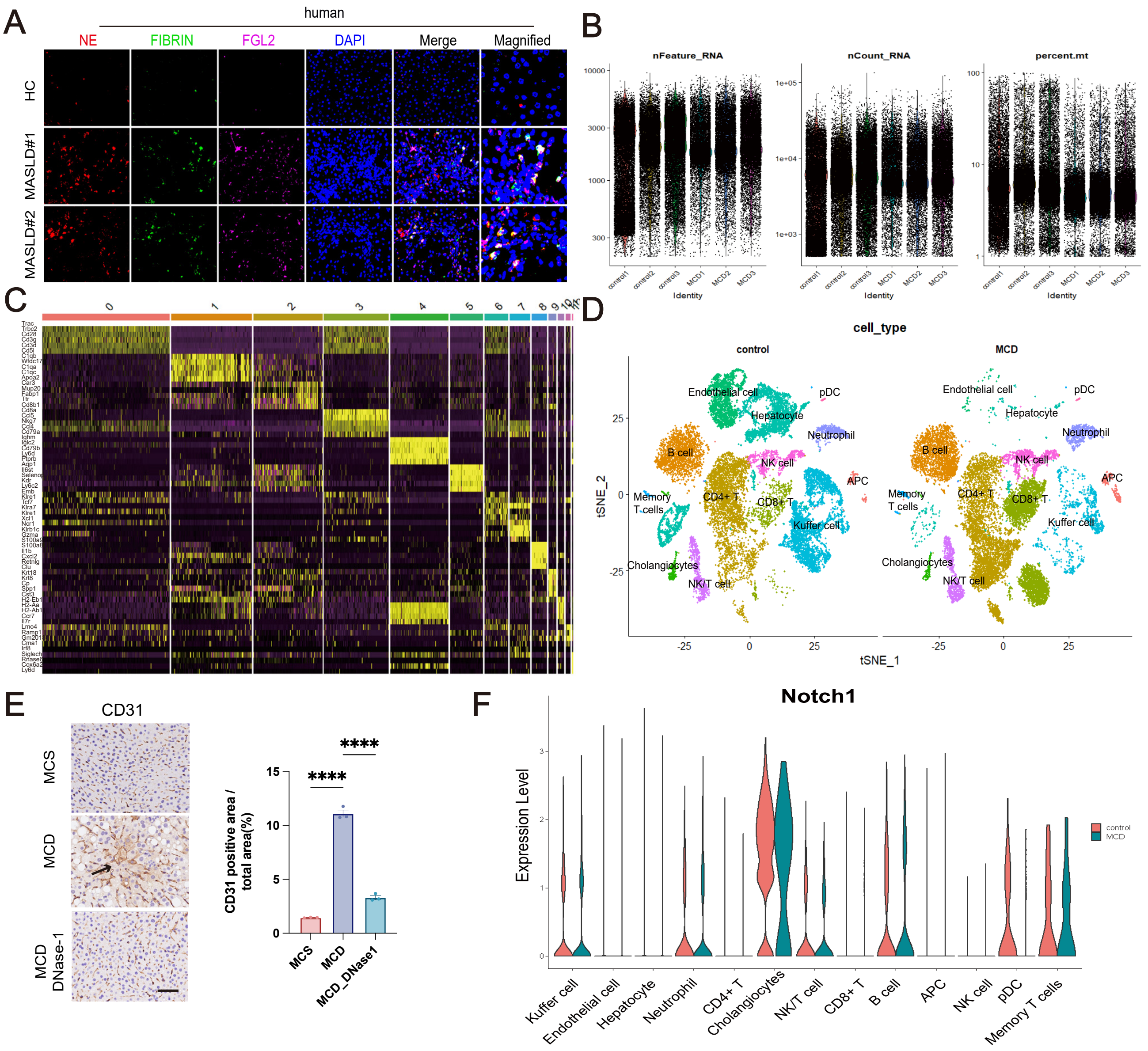
A
human
NE
FIBRIN
FGL2
DAPI
Merge
Magnified
HC
MASLD#1
MASLD#2
B
nFeature_RNA
nCount_RNA
percent.mt
10000
3000
1000
300
1e+05
1e+04
1e+03
100
10
1
control1
control2
control3
MCD1
MCD2
MCD3
Identity
C
D
cell_type
control
MCD
Endothelial cell
pDC
Hepatocyte
Neutrophil
B cell
NK cell
APC
Memory T cells
CD4+ T
CD8+ T
Kuffer cell
Cholangiocytes
NK/T cell
tSNE_2
tSNE_1
E
CD31
MCS
MCD
MCD DNase-1
CD31 positive area / total area(%)
****
****
MCD_DNase1
F
Notch1
Expression Level
control
MCD
Kuffer cell
Endothelial cell
Hepatocyte
Neutrophil
CD4+ T
Cholangiocytes
NK/T cell
CD8+ T
B cell
APC
NK cell
pDC
Memory T cells
