# Supplementary material for: FGL2‐HDAC11 Drives Immunothrombosis via NETs‐Mediated Endothelial Capillarization in MASLD Fibrosis
Source: Adv Sci (Weinh). 2026 May 10;13(44):e22985. doi: 10.1002/advs.202522985 (PMC13335973; doi:10.1002/advs.202522985)
Supplement: Supplementary file 2 [file ADVS-13-e22985-s002.pdf]

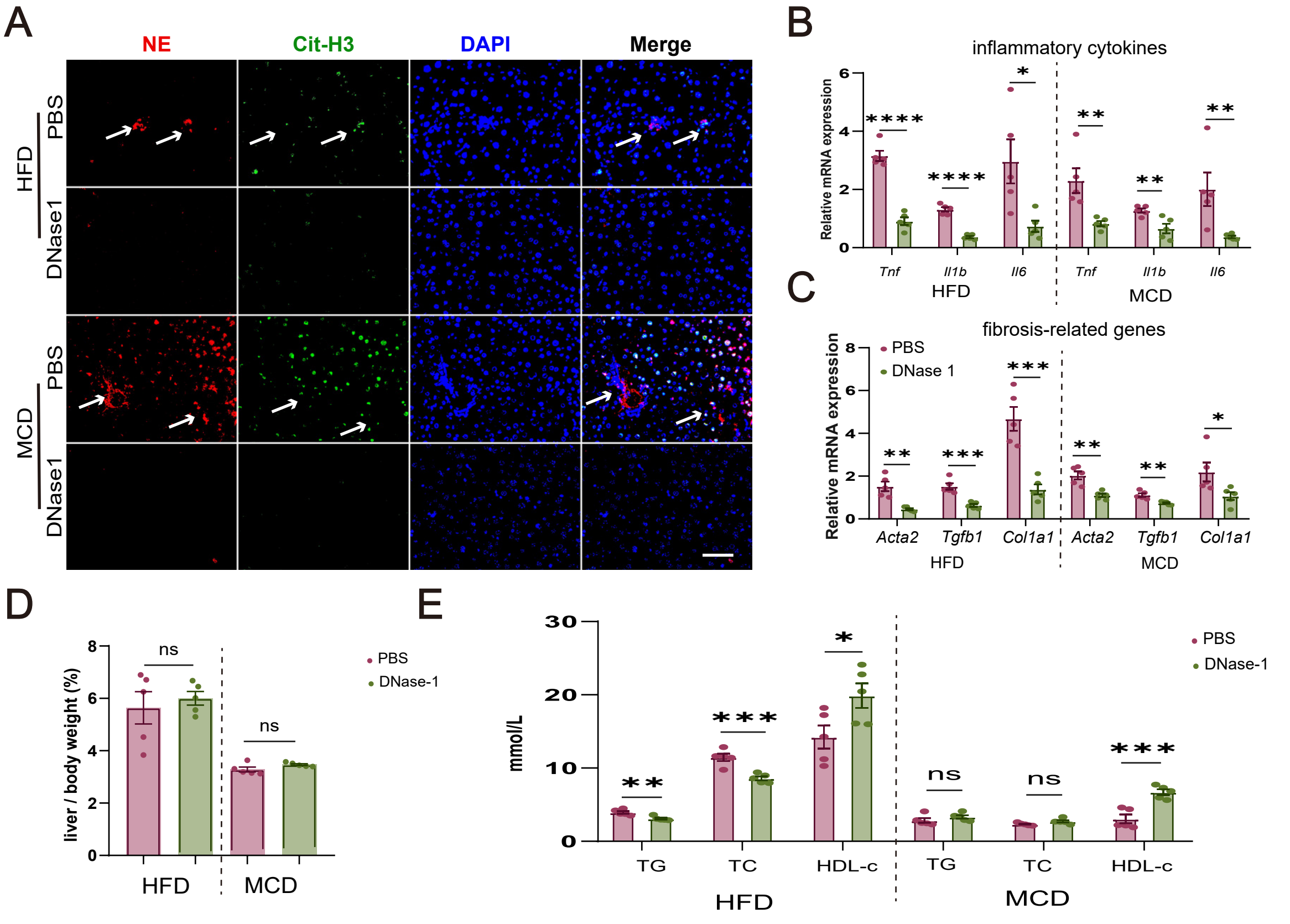

A
NE
Cit-H3
DAPI
Merge
HFD
PBS
DNase1
MCD
PBS
DNase1
B
inflammatory cytokines
Relative mRNA expression
Tnf
Il1b
Il6
Tnf
Il1b
Il6
HFD
MCD
C
fibrosis-related genes
PBS
DNase 1
Relative mRNA expression
Acta2
Tgfb1
Col1a1
Acta2
Tgfb1
Col1a1
HFD
MCD
D
liver / body weight (%)
ns
ns
PBS
DNase-1
HFD
MCD
E
mmol/L
PBS
DNase-1
ns
ns
TG
TC
HDL-c
TG
TC
HDL-c
HFD
MCD
