# Supplementary material for: FGL2‐HDAC11 Drives Immunothrombosis via NETs‐Mediated Endothelial Capillarization in MASLD Fibrosis
Source: Adv Sci (Weinh). 2026 May 10;13(44):e22985. doi: 10.1002/advs.202522985 (PMC13335973; doi:10.1002/advs.202522985)
Supplement: Supplementary file 3 [file ADVS-13-e22985-s003.pdf]

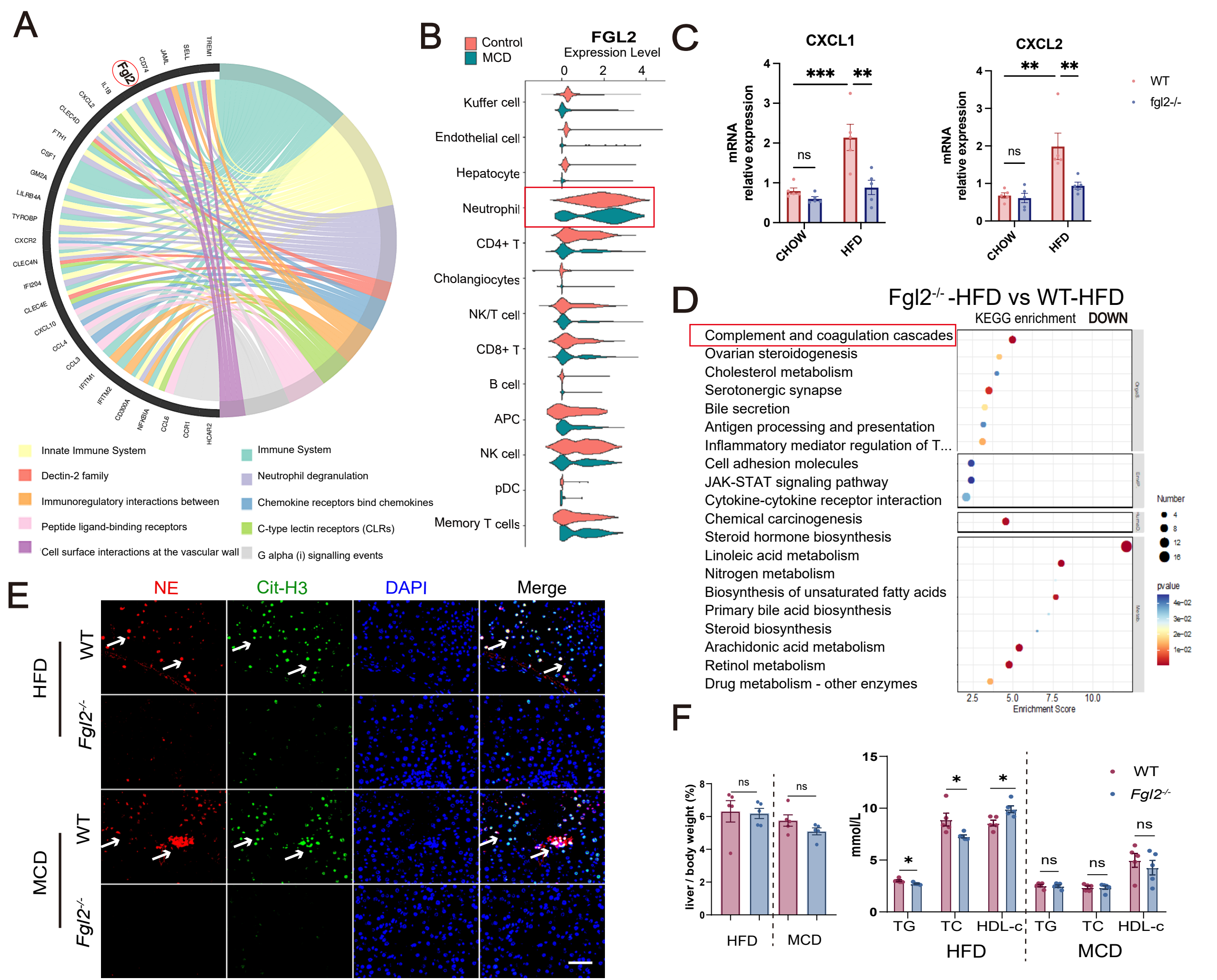

A
Fgl2
Innate Immune System
Dectin-2 family
Immunoregulatory interactions between
Peptide ligand-binding receptors
Cell surface interactions at the vascular wall
Immune System
Neutrophil degranulation
Chemokine receptors bind chemokines
C-type lectin receptors (CLRs)
G alpha (i) signalling events
B
Control
MCD
FGL2
Expression Level
Kuffer cell
Endothelial cell
Hepatocyte
Neutrophil
CD4+ T
Cholangiocytes
NK/T cell
CD8+ T
B cell
APC
NK cell
pDC
Memory T cells
C
CXCL1
CXCL2
mRNA relative expression
CHOW
HFD
WT
fgl2-/-
D
Fgl2-/- -HFD vs WT-HFD
KEGG enrichment DOWN
Complement and coagulation cascades
Ovarian steroidogenesis
Cholesterol metabolism
Serotonergic synapse
Bile secretion
Antigen processing and presentation
Inflammatory mediator regulation of T...
Cell adhesion molecules
JAK-STAT signaling pathway
Cytokine-cytokine receptor interaction
Chemical carcinogenesis
Steroid hormone biosynthesis
Linoleic acid metabolism
Nitrogen metabolism
Biosynthesis of unsaturated fatty acids
Primary bile acid biosynthesis
Steroid biosynthesis
Arachidonic acid metabolism
Retinol metabolism
Drug metabolism - other enzymes
Enrichment Score
Number
pvalue
E
NE
Cit-H3
DAPI
Merge
HFD
MCD
WT
Fgl2-/-
F
liver / body weight (%)
mmol/L
TG
TC
HDL-c
HFD
MCD
WT
Fgl2-/-
