# Supplementary material for: FGL2‐HDAC11 Drives Immunothrombosis via NETs‐Mediated Endothelial Capillarization in MASLD Fibrosis
Source: Adv Sci (Weinh). 2026 May 10;13(44):e22985. doi: 10.1002/advs.202522985 (PMC13335973; doi:10.1002/advs.202522985)
Supplement: Supplementary file 4 [file ADVS-13-e22985-s004.pdf]

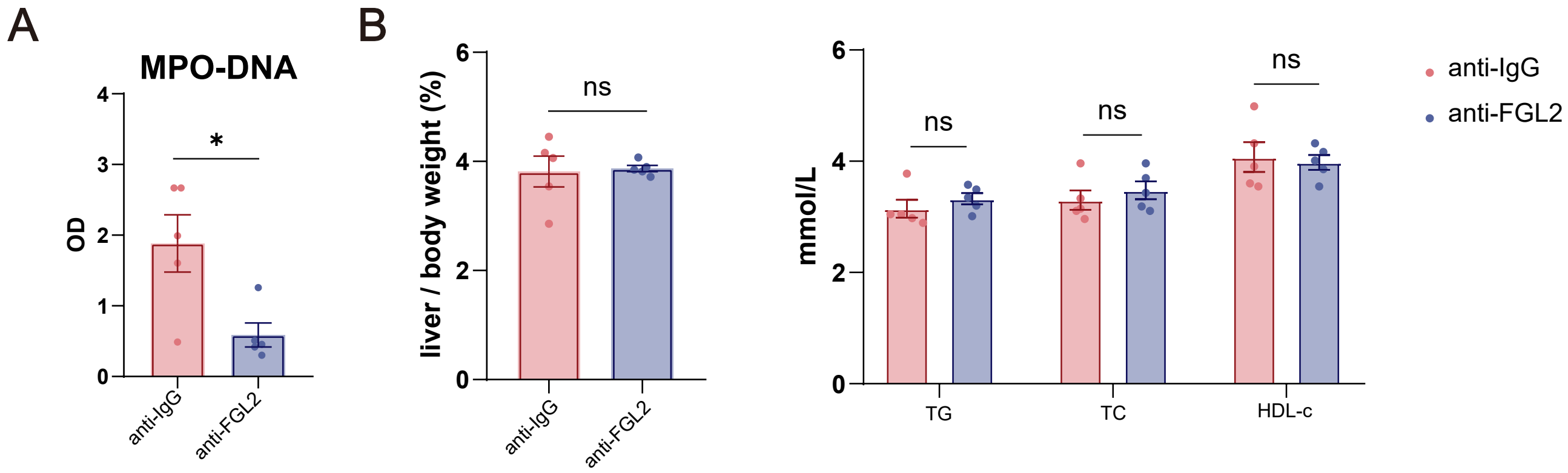

A
MPO-DNA
OD
0
1
2
3
4
*
anti-IgG
anti-FGL2
B
liver / body weight (%)
0
2
4
6
ns
anti-IgG
anti-FGL2
mmol/L
0
2
4
6
ns
ns
ns
TG
TC
HDL-c
anti-IgG
anti-FGL2
