# Supplementary material for: FGL2‐HDAC11 Drives Immunothrombosis via NETs‐Mediated Endothelial Capillarization in MASLD Fibrosis
Source: Adv Sci (Weinh). 2026 May 10;13(44):e22985. doi: 10.1002/advs.202522985 (PMC13335973; doi:10.1002/advs.202522985)
Supplement: Supplementary file 5 [file ADVS-13-e22985-s005.pdf]

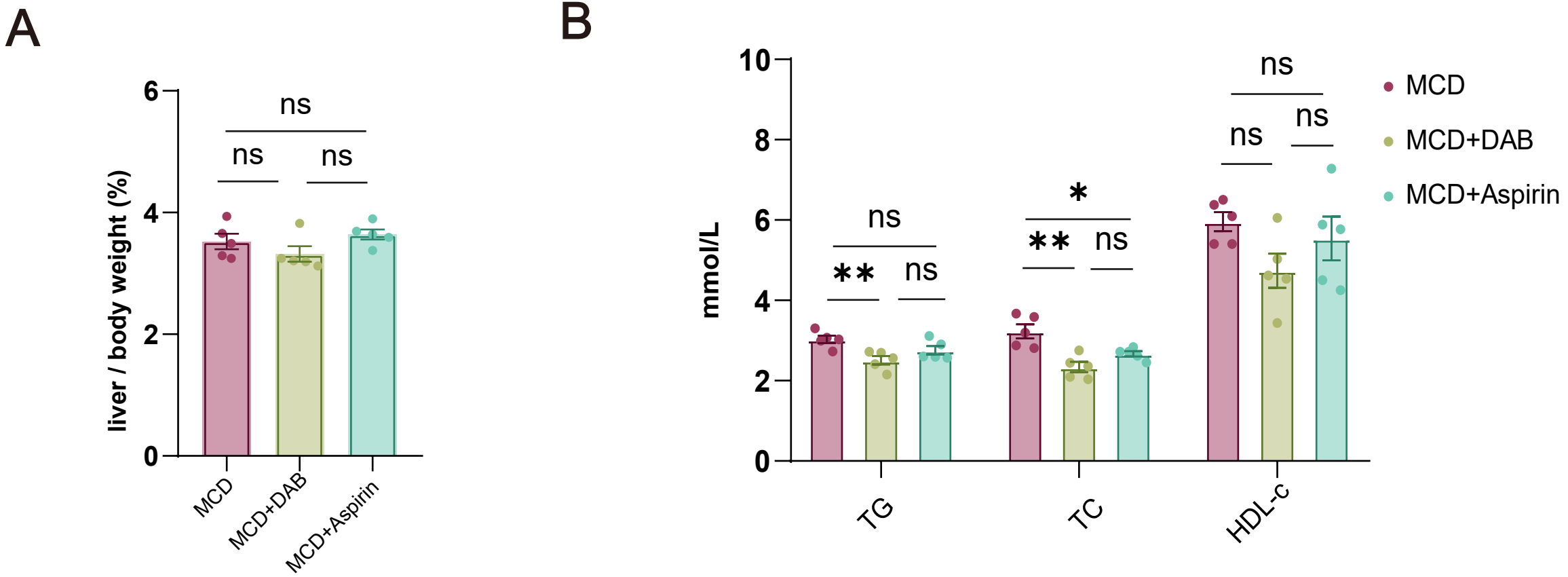
A
liver / body weight (%)
0
2
4
6
ns
ns
ns
MCD
MCD+DAB
MCD+Aspirin
B
mmol/L
0
2
4
6
8
10
ns
**
ns
*
**
ns
ns
ns
ns
TG
TC
HDL-c
MCD
MCD+DAB
MCD+Aspirin
