# Supplementary material for: FGL2‐HDAC11 Drives Immunothrombosis via NETs‐Mediated Endothelial Capillarization in MASLD Fibrosis
Source: Adv Sci (Weinh). 2026 May 10:e22985. Online ahead of print. doi: 10.1002/advs.202522985 (PMC13335973; doi:10.1002/advs.202522985)
Supplement: Supplementary file 1 — Supporting File 1: advs75659‐sup‐0001‐SuppMat.docx. [file ADVS-9999-e22985-s004.docx]

**Supplementary materials and methods**

**Patient sample collection**

A total of 14 patients with MASLD and 10 control subjects were enrolled in this study, and their clinical characteristics are summarized in Table S1. All MASLD patients were diagnosed with hepatic steatosis via ultrasound and/or liver histology and met the diagnostic criteria of having at least one cardiometabolic risk factor as specified in international MASLD guidelines [1]. Subjects were excluded if they had evidence of secondary causes of steatosis or alternative liver diseases, including excessive alcohol intake, drug-induced or genetically determined hepatic steatosis, chronic viral hepatitis (hepatitis B or C), autoimmune liver disease, primary liver tumor or severe extrahepatic disorders.

MASLD patients were further stratified into two subgroups based on the non-alcoholic fatty liver disease activity score (NAS) [2], the NAS < 3 group and the NAS ≥ 3 group. Plasma samples were collected from physical examination center, while liver samples were obtained from patients undergoing partial hepatectomy. The research was conducted in accordance with both the Declarations of Helsinki and Istanbul. All participants provided written informed consent, and all procedures were approved by the Clinical Trial Ethics Committee of Huazhong University of Science and Technology (2024S091).

**Animal Housing and Diet-Induced MASLD Models**

Mice were maintained under specific pathogen-free (SPF) conditions at the Animal Experiment Center of Tongji Hospital. *Fgl2^-/-^* mice with a C57BL/6 genetic background were purchased from Shanghai Model Organisms Center, Inc. (Shanghai, China). Eight-week-old wild-type (WT) littermates were randomly assigned to experimental and control groups. Two diet-induced MASLD models were established: 1. Mice were fed either a 60 kcal% high-fat diet (HFD) (MD12033, Medicine, Jiangsu, China) for 36 weeks or a methionine/choline-deficient (MCD) diet (MD12051, Medicine, Jiangsu, China) for 5 weeks. Control groups received either a standard chow diet (MD17111, Medicine, Jiangsu, China) or a methionine/choline-sufficient (MCS) diet (MD12052, Medicine, Jiangsu, China). All animal experiments were conducted in compliance with the protocols approved by the Tongji Hospital Animal Ethics Committee (TJH-202311018).

**Animal treatment**

NETs were depleted by intraperitoneal injection of DNase-1 (Solarbio, Beijing, China) at a dosage of 2.5 mg/kg, administered three times per week. For MCD-fed mice, DNase-1 treatment initiated at week 2 and continued until week 5. For HFD-fed mice, DNase-1 treatment was administered from week 8 to week 36. In some experiments, neutrophils (4×10^6 cells) isolated from WT or *fgl2^-/-^* mice were transferred into *fgl2^-/-^* mice fed an MCD diet. Transfers were performed weekly for 3 weeks after the start of MCD diet [3].

**Liver Leukocytes Preparation**

Liver leukocytes were isolated using a a previously established protocol with minor modifications [3]. Briefly, fresh liver tissues were digested with collagenase IV to disperse cell aggregates. The digested mixture was filtered through a 100-μm cell strainer to remove tissue debris. The resulting cell pellet was resuspended in 10 mL of 35% Percoll solution, and leukocytes were isolated via density gradient centrifugation. All procedures were performed on ice under sterile conditions to maintain cell viability and prevent nonspecific cell activation. Isolated leukocytes were used for single-cell RNA sequencing (scRNA-seq).

**scRNA-seq Data Acquisition and Processing**

Liver leukocytes were isolated from mice fed a HFD for 36 weeks. After verifying cell viability (>90% via trypan blue exclusion assay), viable cells were subjected to scRNA-seq using NovaSeq 6000 platform (Illumina, San Diego, CA, USA) following standard leukocyte scRNA-seq protocols. scRNA-seq data for chow-fed control mice were retrieved from the Gene Expression Omnibus (GEO) database under accession number GSE129516. Data for MCD diet-fed mice were obtained from GEO accession number GSE166178.

Data preprocessing was performed using Seurat V4 software [4]. Firstly, cells with <500 detected genes or >20% mitochondrial reads (indicative of apoptosis or low integrity) were excluded. Then genes expressed in <10 cells (low-confidence transcripts) were filtered out. Read alignment and gene quantification were conducted using Cell Ranger software [5]. Count matrices were normalized via SCTransform [6], and highly variable genes were selected for downstream analysis.

Dimensionality reduction was performed via principal component analysis (PCA). Cell clustering was conducted using the Louvain algorithm based on PCA results [7], and visualized via t-distributed stochastic neighbor embedding (t-SNE) or Uniform Manifold Approximation and Projection (umap). Cell types were annotated by matching the high-expression gene profile of each cluster to known cell type-specific gene signatures.

**Hepatic whole transcriptome sequencing**

Total RNA was extracted from liver tissues using Trizol reagent (15596026, Invitrogen, USA). RNA quality was assessed using an Agilent 2100 Bioanalyzer (Agilent Technologies, Santa Clara, USA), and RNA concentration was quantified using a NanoDrop 2000 spectrophotometer (Thermo Scientific, USA). Whole transcriptome sequencing libraries were constructed as follows: Firstly, 200 ng of total RNA was used as input for the Illumina TruSeq Stranded mRNA Library Prep Kit, following the manufacturer’s instructions; then the samples were indexed using Illumina TruSeq RNA Single Indexes; lastly, library quality was verified via Agilent 2100 Bioanalyzer, and library concentration was quantified via Qubit fluorometric quantification (Thermo Fisher). Libraries were sequenced on an Illumina NovaSeq 6000 platform to generate 150 bp paired-end reads. Raw sequencing data (fastq format) were processed using Trimmomatic to remove low-quality reads and obtain clean reads. Clean reads were aligned to the mouse reference genome using HISAT2. Fragments per kilobase of transcript per million mapped reads (FPKM) for each gene were calculated using Cufflinks, and read counts were obtained via HTSeq-count. Differential expression analysis was performed using the DESeq (2012) R package. Genes with a p-value < 0.05 and fold change > 2 or < 0.5 were defined as differentially expressed genes (DEGs). Hierarchical cluster analysis was conducted to visualize DEG expression patterns across groups and samples. Gene Ontology (GO) enrichment analysis and Kyoto Encyclopedia of Genes and Genomes (KEGG) pathway enrichment analysis of DEGs were performed using R software based on the hypergeometric distribution.

**Primary neutrophil isolation**

Bone marrow neutrophils were isolated via density gradient centrifugation combined with magnetic bead sorting as previously described [3]. Briefly, bone marrow cells were flushed from femur and tibia of mice. Cells were subjected to preliminary separation using histopaque 1119 and 1077 (Sigma, St. Louis, MO), and neutrophils were further purified using MACS microbead (Miltenyi Biotec, San Diego, CA). Neutrophil viability and purity were confirmed to exceed 90% via flow cytometry.

**NETs detection**

For histological analysis, NETs were stained with either Cit-H3 and NE using immunofluorescence. The colocalization of two NETs biomarkers were defined as NETs formation [8]. The level of MPO–DNA, a recognized circulating biomarker of NETs, was detected using the ELISA capture method as previously reported [9]. Briefly, plasma or supernatant mixed with peroxidase-labeled anti-DNA monoclonal antibody (component 2 of the Cell Death Detection ELISA kit, Roche, Basel, Switzerland) were added to MPO-coated 96 wells, followed by an incubation at room temperature for 2 hours. Subsequently, peroxidase substrate was added, and the plates were incubated at 37°C for 30 minutes. The optical density was measured at 450 nm.

For morphological observation, neutrophils were seeded on coverslips precoated with poly-lysine and stimulated with different treatments. Immediately after stimulation, 5μM SYTOX Green was added to the cells to detect extracellular DNA [3, 10]. For extracellular DNA detection, neutrophils were seeded in 96-well, black, clear-bottom non-tissue culture plate (Costar) and incubated with different treatments. Meanwhile, 500nM SYTOX Green were added to bind extracellular DNA. Fluorescence was then detected using a fluorescence microplate reader (Excitation: 480nm; Emission: 520nm) [11].

**Cell culture and treatment**

For in vitro studies, neutrophils were seeded in 12-well plates and stimulated with palmitic acid (PA, 200μM). The cells were then stimulated either an HDAC11 antagonist (1μM) [12] or anti-FGL2 N-terminal monoclonal antibody for 3 hours. The stimulated cells were collected for further analyses.

**Co-immunoprecipitation**

Bone marrow-derived neutrophils (BM-neutrophils) from WT mice were stimulated with PA for 3 hours. After cell lysis, total protein was extracted, and samples were precleared with protein A/G magnetic beads. The samples were then incubated with Fgl2 antibody overnight at 4°C, followed by a 4-hour incubation with magnetic beads at the same temperature. The immunoprecipitated beads were heated at 100°C for 8 minutes in lysis buffer. Western blotting was used to detect Fgl2, Hdac11, and mouse IgG1 isotype control antibodies.

**Immunohistochemistry (IHC) and** **Immunofluorescence (IF)**

Liver specimens were fixed overnight in 4% paraformaldehyde and embedded in paraffin for further detection. The sections were stained with hematoxylin and eosin (H&E), Masson’s trichrome, or various primary antibodies (anti-αSMA, anti-fibrinogen, anti-CD31). The sections were observed under a microscope (CX22, OLYMPUS, Japan). For IHC analysis, 5–8 different positive microscopic fields from three slides were selected for further analysis. Mean fluorescence intensity and colocalization were analyzed using ImageJ software.

For IF staining, paraffin-embedded mouse liver tissues were subjected to antigen retrieval. After washing and blocking, the slides were incubated with the following primary antibodies: anti-neutrophil elastase, anti-CitH3, anti-fibrinogen, anti-fgl2, anti-LY6G, anti-Hdac11, anti-CD31. Subsequently, species-specific secondary antibodies coupled with Alexa Fluor Dyes (Servicebio, China) were used, and DNA was stained with DAPI (G1012, Servicebio, China). The sections were observed under a fluorescence microscope (BX53, OLYMPUS, Japan). Detailed information about the antibodies (such as dilution ratios, catalog numbers, manufacturers, etc.) can be found in Supplementary Table 2.

**Western Blot analysis**

Protein samples were extracted from liver homogenates or cell lysates and quantified using a bicinchoninic acid (BCA) protein assay kit (AR1189, BOSTER, China). The samples were then separated on polyacrylamide gels (NewFlash Protein AnyKD PAGE, 8012011; Dakewe Biotech, China) and transferred to PVDF membranes. After blocking and washing, the membranes were incubated with primary antibodies overnight (PAD4, Cit-H3, anti-fgl2, Hdac11, Acetylated-H3 (Acel-H3), Histone 3, β-actin, PCNA). After washing with TBST, the membranes were incubated with secondary antibodies for 40 minutes at room temperature. Proteins were visualized using a chemiluminescence system (ChemiDoc XRS+, Bio-Rad), and their integrated optical densities were quantified using Image Lab software. Detailed information about the antibodies (such as dilution ratios, catalog numbers, manufacturers, etc.) can be found in Supplementary Table 2.

**Quantitative Polymerase Chain Reaction (qPCR)**

Total RNA was extracted from liver tissues using RNAiso Plus (9109, Takara, Japan). Complementary DNA (cDNA) was synthesized from 2 μg of RNA using the ReverTraAce@qPCR RT kit (TOYOBO, Osaka, Japan) according to the manufacturer’s instructions. SYBR Green Real-Time Polymerase Chain Reaction Master Mix (TOYOBO, Osaka, Japan) was used to detect the expression of target genes using a real-time polymerase chain reaction system (CXF96; Bio-Rad, Hercules, CA). The primer sequences are provided in Supplementary Table 3.

**ELISA**

Plasma levels of TAT (human: ab108907, abcam, USA; mouse: NBP2-75962, NOVUS, USA), C3a (human: NBP2-66755, NOVUS, USA; mouse: NBP2-70037, NOVUS, USA) and C5a (human: ab193695, abcam, USA; mouse: ab193718, abcam, USA) were detected using commercial kits according to the manufacturer’s instructions. The expression level of alanine aminotransferase (ALT), aspartate aminotransferase (AST), triglyceride (TG), cholesterol (TC), and high-density lipoprotein cholesterol (HDL-C) were analyzed using a spectrophotometer (Bio-Rad) according to manufacturer's instructions (Nanjing Jiancheng; TG, A110-1-1; TC, A111-1-1; HDL-C, A112-1-1; ALT, C009-2-1; AST, C010-2-1). APTT and PT were measured using sodium citrate-anticoagulated plasma. The clotting times were recorded on a coagulation analyzer according to the manufacturer’s instructions.

**Laser speckle contrast imaging**

Hepatic blood perfusion was monitored using a laser speckle blood flow imaging system (JVS-RLS-PRO) in anesthetized mice via intra-operative imaging. After laparotomy, the left liver lobe was gently exposed and stabilized to minimize motion artifacts, and the procedure was performed in strict accordance with previously validated protocols [13]. Through this system, the region of interest was illuminated with a 780 nm divergent laser beam, creating a speckle pattern over the illuminated area. The speckle pattern in the illuminated area was monitored using an sCMOS camera (resolution: 1472×1104 pixels) at an acquisition rate of 10 fps. The acquired signal was converted into a two-dimensional blood perfusion map to reflect hepatic blood perfusion information.

**Scanning electron microscopy**

Liver tissue preparation and SEM were carried out as previously described [14]. Briefly, liver tissues were fixed in situ by portal vein perfusion with normal saline, followed by Trump’s fixative (4% formaldehyde, 1% glutaraldehyde in PBS, pH 7.2). After overnight fixation at 4°C, the livers were cut into 2 mm² pieces, dehydrated in a graded ethanol series, and dried. Samples were sputter-coated with platinum/palladium and imaged using a Hitachi S-4700 SEM. Fenestrae in liver sinusoidal endothelial cells (LSECs) were quantified in five random fields per mouse using ImageJ, as previously described.

**Statistical analysis**

Data are presented as mean ± standard error of the mean (SEM). Comparisons between two groups were performed using Student’s t-test. For comparisons among three or more groups, one-way ANOVA followed by Tukey’s multiple comparisons test was used. All parametric tests were preceded by checks for normality and homogeneity of variance. Exact p-values are reported whenever feasible. Statistical analyses were conducted using GraphPad Prism 9, with p < 0.05 considered significant.

**Supplemental Figures**

**Figure S1. Effects of dabigatran and aspirin treatment on lipid metabolism in MASLD progression.** **(A)** Liver-to-body weight ratio in MCD diet-fed mice treated with either dabigatran or aspirin. **(B)** Effects of anticoagulant therapy on lipid metabolism parameters in MASLD mice. N=5 in each group, **p* < .05, ***p* < .01, ****p* < .001, and *****p* < .0001.

**Figure S2. NETs depletion improved metabolic dysregulation in MASLD. (A)** Quantification of hepatic NETs (NE, red; Cit-H3, green) following DNase-1 treatment (n=3 in each group). **(B)** Hepatic expression profiles of inflammatory cytokines (*Tnf-α, il-1β, Il-*6) post-intervention (n=5 in each group). **(C)** mRNA expression of fibrogenic markers (*Acta2, Tgfb1, Col1a1*) following DNase-1 treatment (n=5 in each group). **(D)** Changes in liver-to-body weight ratio post-intervention (n=5 in each group). **(E)** Effects of DNase-1 treatment on plasma lipid metabolism parameters (n = 5 in each group). Scale bar: 50 μm, **p* < .05, ***p* < .01, ****p* < .001, and *****p* < .0001.

**Figure S3. FGL2 exacerbates MASLD progression by mediating NETs release and subsequent coagulation dysregulation.** **(A)** Functional enrichment of FGL2+ neutrophil cluster (Cluster 0) from scRNA-seq data. **(B)** Violin plot from single-cell sequencing showing FGL2 expression across different cell subpopulations in both WT and MCD-fed mice. **(C)** The mRNA expression levels of *Cxcl1* and *Cxcl2* in the livers of WT and *fgl2⁻/⁻* mice fed a high-fat diet. **(D)** KEGG analysis of differentially expressed genes in *fgl2^-/-^* and WT mice fed an HFD diet. **(E)** Immunofluorescence detection of hepatic NETs release (NE, red; Cit-H3, green) in *fgl2^-/-^* and WT mice (n=3 in each group). **(F)** Liver-to-body weight ratios across genotypes in *fgl2^-/-^* and WT mice. Plasma lipid metabolic profiles in *fgl2^-/-^* and WT mice (n=5 in each group). Scale bar = 50 μm, **p* < .05, ***p* < .01, ****p* < .001, and *****p* < .0001.

**Figure S4. scRNA-seq Quality Control and Cell Subpopulation Characterization of Hepatic nonparenchymal cells.** **(A)** Co-immunofluorescence staining of liver tissues from humans showing NETs (NE; green), fibrin (green), and FGL2 (magenta). **(B)** Quality control metrics for scRNA-seq: number of detected features per cell (nFeature), total reads per cell (nCount), and mitochondrial gene percentage per cell (percent MT). These metrics filtered low-quality cells to ensure data reliability. **(C)** Heatmap of highly expressed genes (marker genes) for each cell cluster. Columns represent cell clusters, and rows represent marker genes; color intensity indicates gene expression level. This heatmap was used to preliminarily infer the identity of each cell cluster. **(D)** t-SNE plot of qualified cells. Visualizes cell clustering, reflecting cell composition differences between groups. **(E)** Perisinusoidal capillarization in DNase-1-treated MCD mice. (F-G) Violin plots of Notch1 **(F)** and FGL2 **(G)** expression in each cell subpopulation. Used to compare gene expression across subpopulations. Scale bar: 50 μm, n= 3-5 in each group, **p* < .05, ***p* < .01, ****p* < .001, and *****p* < .0001.

**Figure S5. FGL2 monoclonal antibody exhibits minimal impact on lipid metabolism.** **(A)** Quantitative assessment of circulating NETs markers (MPO-DNA complexes). **(B)** Liver-to-body weight ratio in mice following treatment with FGL2 monoclonal antibody. Changes in blood lipid metabolism after administration of FGL2 monoclonal antibody. **p* < .05, ***p* < .01, ****p* < .001, and *****p* < .0001.

**Supplemental Tables**

**Supplemental Table 1.** The clinical features of MASLD and controls.

|  | **Control（n= 10)** | **MASLD（n= 14)** | *P* |
| --- | --- | --- | --- |
| Age (years) | 50±5 | 50±4 | 0.9544 |
| Sex, male (%) | 4（40%） | 5（35.71%） | >0.9999 |
| Body mass index (kg/m2) | 22.55±0.85 | 24.48±0.73 | 0.1005 |
| Diabetes (%) | 1（10%） | 5（35.71%） | 0.3408 |
| ALT (U/L) | 16.4±4.26 | 16±2.08 | 0.9274 |
| AST (U/L) | 22.7±3.00 | 19.07±0.94 | 0.2007 |
| WBC（10^9/L) | 5.71±0.62 | 6.22±0.55 | 0.5487 |
| Neutrophil（10^9/L) | 3.40±0.53 | 3.49±0.34 | 0.8729 |
| PLT (10^9/L) | 221.3±21.64 | 241±18.49 | 0.497 |
| PT(s) | 13.07±0.16 | 13.19±0.18 | 0.6488 |
| APTT(s) | 36.47±0.98 | 35.81±0.97 | 0.6483 |
| TT(s) | 17.31±0.27 | 17.43±0.27 | 0.7642 |
| Fibrinogen(g/L） | 3.43±0.23 | 3.17±0.20 | 0.4033 |
| D-Dimer(ug/ml) | 0.8±0.85 | 0.86±0.27 | 0.8819 |

**Supplementary Table 2.** Antibodies used in this study.

| Antibody Name | Application | Dilution | Catalog Number | Manufacturer | Source Region |
| --- | --- | --- | --- | --- | --- |
| Fgl2 antibody | Co-IP, WB, IF | 1:100(IF); 1:1000(WB) | H00010875-M01 | Abnova | China |
| Hdac11 antibody | Co-IP, WB, IF | 1:100(IF); 1:1000 (WB) | sc-390737 | Santa Cruz | USA |
| Mouse IgG1 isotype control | Co-IP | 1:1000 (WB) | 5415 | CST | USA |
| Anti-αSMA | IHC | 1:100 | 19245 | CST | USA |
| Anti-fibrinogen | IHC | 1:200 | ab34269 | Abcam | USA |
| Anti-CD31 | IF, IHC | 1:200 | ab182981 | Abcam | USA |
| Anti-neutrophil elastase (NE) | IF | 1:100 | ab68672 | Abcam | USA |
| Anti-CitH3 | IF, WB | 1:100(IF); 1:1000 (WB) | ab5103 | Abcam | USA |
| Anti-LY6G | IF | 1:100 | ab238132 | Abcam | USA |
| PAD4 antibody | WB | 1:1000 | 17373-1-AP | Proteintech | USA |
| Acetylated-H3 (Acel-H3) antibody | WB | 1:1000 | 06-599 | Millipore | USA |
| Histone3 antibody | WB | 1:1000 | 4499S | CST | USA |
| β-actin antibody | WB | 1:1000 | 4970S | CST | USA |
| PCNA antibody | WB | 1:1000 | 10205-2-AP | Proteintech | USA |

**Supplementary Table 3.** List of all primers used in this study.

| Gene name | Primer | Sequence | |
| --- | --- | --- | --- |
| *Tnf* | forward | 5'-TGGAACTGGCAGAAGAGGCACT -3' | |
|  | reverse | 5'-CCATAGAACTGATGAGAGGGAGGC -3' | |
| *Il1b* | forward | 5'-TCTTGTGTAAAATGAAAGACGGC -3' | |
|  | reverse | 5'-ACTCCACTTTGCTCTTGACTTC -3' | |
| *Il6* | forward | 5'-AGGATACCACTCCCAACAGACCT -3' | |
|  | reverse | 5'-CAAGTGCATCATCGTTGTTCATAC -3' | |
| Acta2 | forward | 5'-TGGCACCACACCTTCTACAA -3' | |
|  | reverse | 5'-CGGAGGCATAGAGGGACA -3' | |
| *Tgfb1* | forward | 5'-CTATGCTAAAGAGGTCACCCG -3' | |
|  | reverse | 5'-ACTGCTTCCCGAATGTCTG -3' | |
| *Col1a1* | forward | 5'-CATAAAGGGTCATCGTGGCT -3' |  |
|  | reverse | 5'-TTGAGTCCGTCTTTGCCAG -3' |  |
| *Piezo1* | forward | 5’-TACGCCGAGGTGTGCTGGAC-3’ |  |
|  | reverse | 5’-GCTGGTGTCGTCTGTCATGCTAC-3’ |  |
| *Notch1* | forward | 5’-GTGTCCCAAAGGCTTCAGC-3’ |  |
|  | reverse | 5’-CGTTCTTGCATGGTGTGCT-3’ |  |
| *Actin* | forward | 5'-GGTCAGAAGGACTCCTATGTGG -3' | |
|  | reverse | 5'-TGTCGTCCCAGTTGGTAACA -3' | |

**References**

1. Rinella, M. E., J. V. Lazarus, V. Ratziu*, et al.* "A Multisociety Delphi Consensus Statement on New Fatty Liver Disease Nomenclature." *J Hepatol* 79, no. 6 (2023): 1542-56. <https://doi.org/10.1016/j.jhep.2023.06.003>.

2. Kleiner, David E., Elizabeth M. Brunt, Mark Van Natta*, et al.* "Design and Validation of a Histological Scoring System for Nonalcoholic Fatty Liver Disease†." *HEPATOLOGY* 41, no. 6 (2005): 1313-21. <https://doi.org/10.1002/hep.20701>.

3. Li, X., Q. Gao, W. Wu*, et al.* "Fgl2-Mcoln3-Autophagy Axis-Triggered Neutrophil Extracellular Traps Exacerbate Liver Injury in Fulminant Viral Hepatitis." *Cell Mol Gastroenterol Hepatol* 14, no. 5 (2022): 1077-101. <https://doi.org/10.1016/j.jcmgh.2022.07.014>.

4. Hao, Y., S. Hao, E. Andersen-Nissen*, et al.* "Integrated Analysis of Multimodal Single-Cell Data." *Cell* 184, no. 13 (2021): 3573-87 e29. <https://doi.org/10.1016/j.cell.2021.04.048>.

5. Zheng, G. X., J. M. Terry, P. Belgrader*, et al.* "Massively Parallel Digital Transcriptional Profiling of Single Cells." *Nat Commun* 8 (2017): 14049. <https://doi.org/10.1038/ncomms14049>.

6. Hafemeister, C., and R. Satija. "Normalization and Variance Stabilization of Single-Cell Rna-Seq Data Using Regularized Negative Binomial Regression." *Genome Biol* 20, no. 1 (2019): 296. <https://doi.org/10.1186/s13059-019-1874-1>.

7. Becht, E., L. McInnes, J. Healy*, et al.* "Dimensionality Reduction for Visualizing Single-Cell Data Using Umap." *Nat Biotechnol* (2018). <https://doi.org/10.1038/nbt.4314>.

8. Papayannopoulos, V. "Neutrophil Extracellular Traps in Immunity and Disease." *Nat Rev Immunol* 18, no. 2 (2018): 134-47. <https://doi.org/10.1038/nri.2017.105>.

9. Donkel, S. J., F. J. Wolters, M. A. Ikram, and M. P. M. de Maat. "Circulating Myeloperoxidase (Mpo)-DNA Complexes as Marker for Neutrophil Extracellular Traps (Nets) Levels and the Association with Cardiovascular Risk Factors in the General Population." *PLoS One* 16, no. 8 (2021): e0253698. <https://doi.org/10.1371/journal.pone.0253698>.

10. Brinkmann V, Reichard U, Goosmann C, Fauler B, Uhlemann Y, Weiss DS, Weinrauch Y, Zychlinsky A. "Neutrophil Extracellular Traps Kill Bacteria." *Science* 303(5663):1532-5. (2004). <https://doi.org/10.1126/science.1092385>.

11. Zuo, Y., S. Yalavarthi, H. Shi*, et al.* "Neutrophil Extracellular Traps in Covid-19." *JCI Insight* 5, no. 11 (2020). <https://doi.org/10.1172/jci.insight.138999>.

12. Huang, J., L. Wang, S. Dahiya*, et al.* "Histone/Protein Deacetylase 11 Targeting Promotes Foxp3+ Treg Function." *Sci Rep* 7, no. 1 (2017): 8626. <https://doi.org/10.1038/s41598-017-09211-3>.

13. Li, C. H., X. L. Ge, K. Pan, P. F. Wang, Y. N. Su, and A. Q. Zhang. "Laser Speckle Contrast Imaging and Oxygen to See for Assessing Microcirculatory Liver Blood Flow Changes Following Different Volumes of Hepatectomy." *Microvasc Res* 110 (2017): 14-23. <https://doi.org/10.1016/j.mvr.2016.11.004>.

14. Guo, Q., K. Furuta, S. Islam*, et al.* "Liver Sinusoidal Endothelial Cell Expressed Vascular Cell Adhesion Molecule 1 Promotes Liver Fibrosis." *Front Immunol* 13 (2022): 983255. <https://doi.org/10.3389/fimmu.2022.983255>.
