# Supplementary figures and images for: FGL2‐HDAC11 Drives Immunothrombosis via NETs‐Mediated Endothelial Capillarization in MASLD Fibrosis
Source: Adv Sci (Weinh). 2026 May 10:e22985. Online ahead of print. doi: 10.1002/advs.202522985 (PMC13335973; doi:10.1002/advs.202522985)

**A**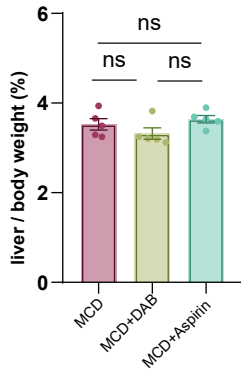**B**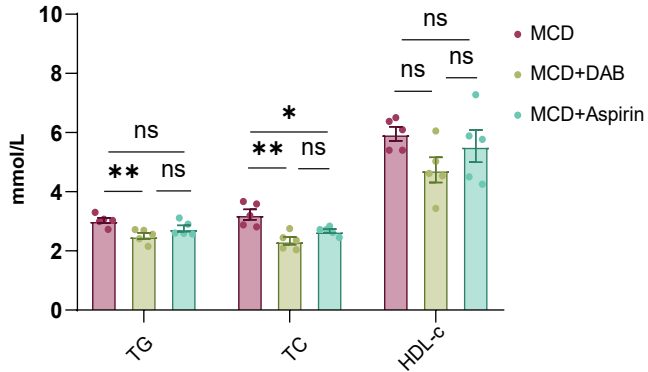

Supplement: Supplementary file 2 — Supporting File 2: advs75659‐sup‐0002‐Figure1.pdf. [file ADVS-9999-e22985-s005.pdf]

**A**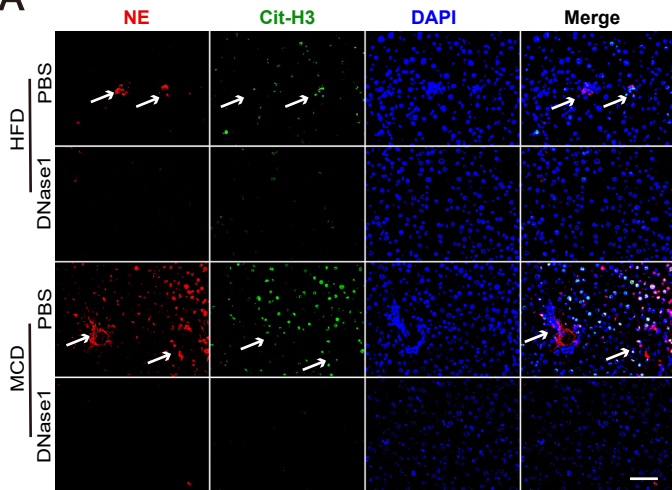**B**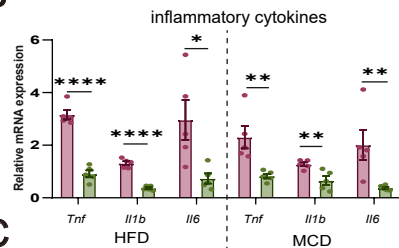**C**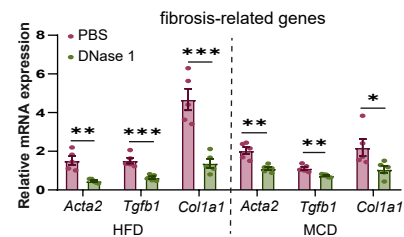**D**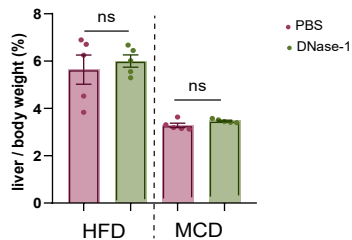**E**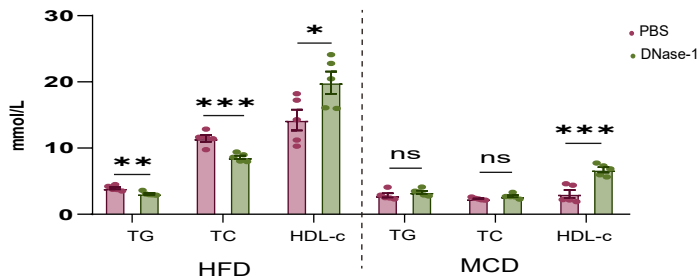

Supplement: Supplementary file 3 — Supporting File 3: advs75659‐sup‐0003‐Figure2.pdf. [file ADVS-9999-e22985-s001.pdf]

A

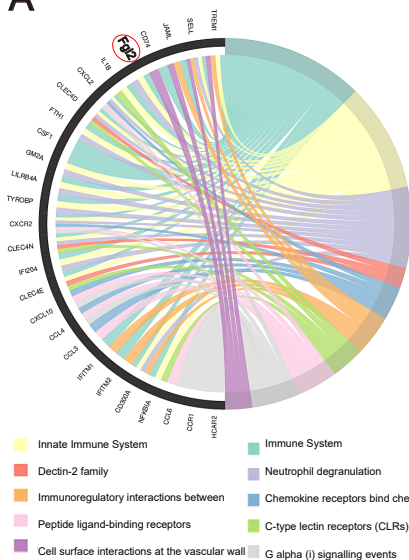

B

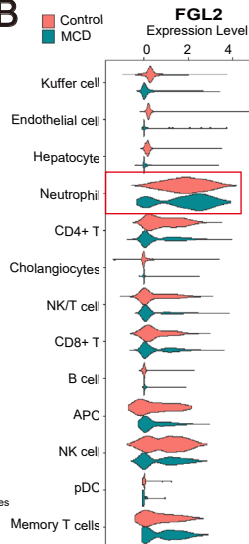

C

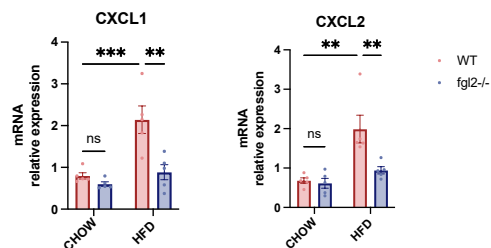

D

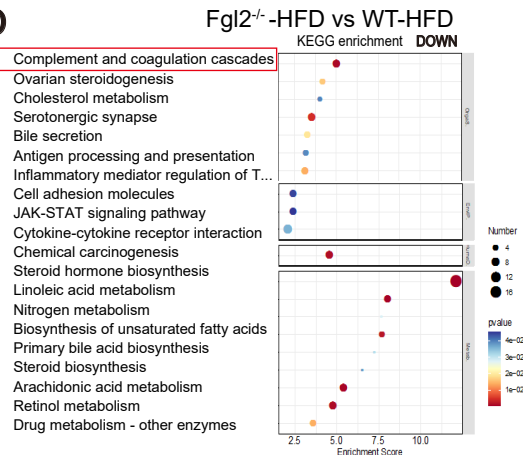

E

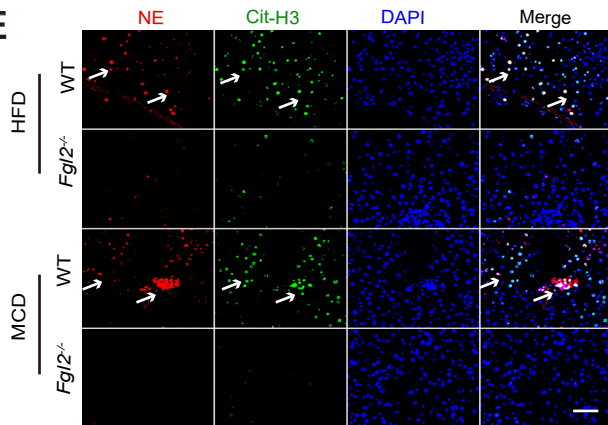

F

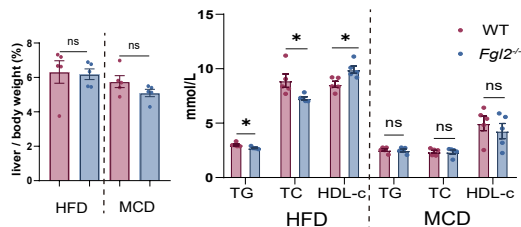

Supplement: Supplementary file 4 — Supporting File 4: advs75659‐sup‐0004‐Figure3.pdf. [file ADVS-9999-e22985-s002.pdf]

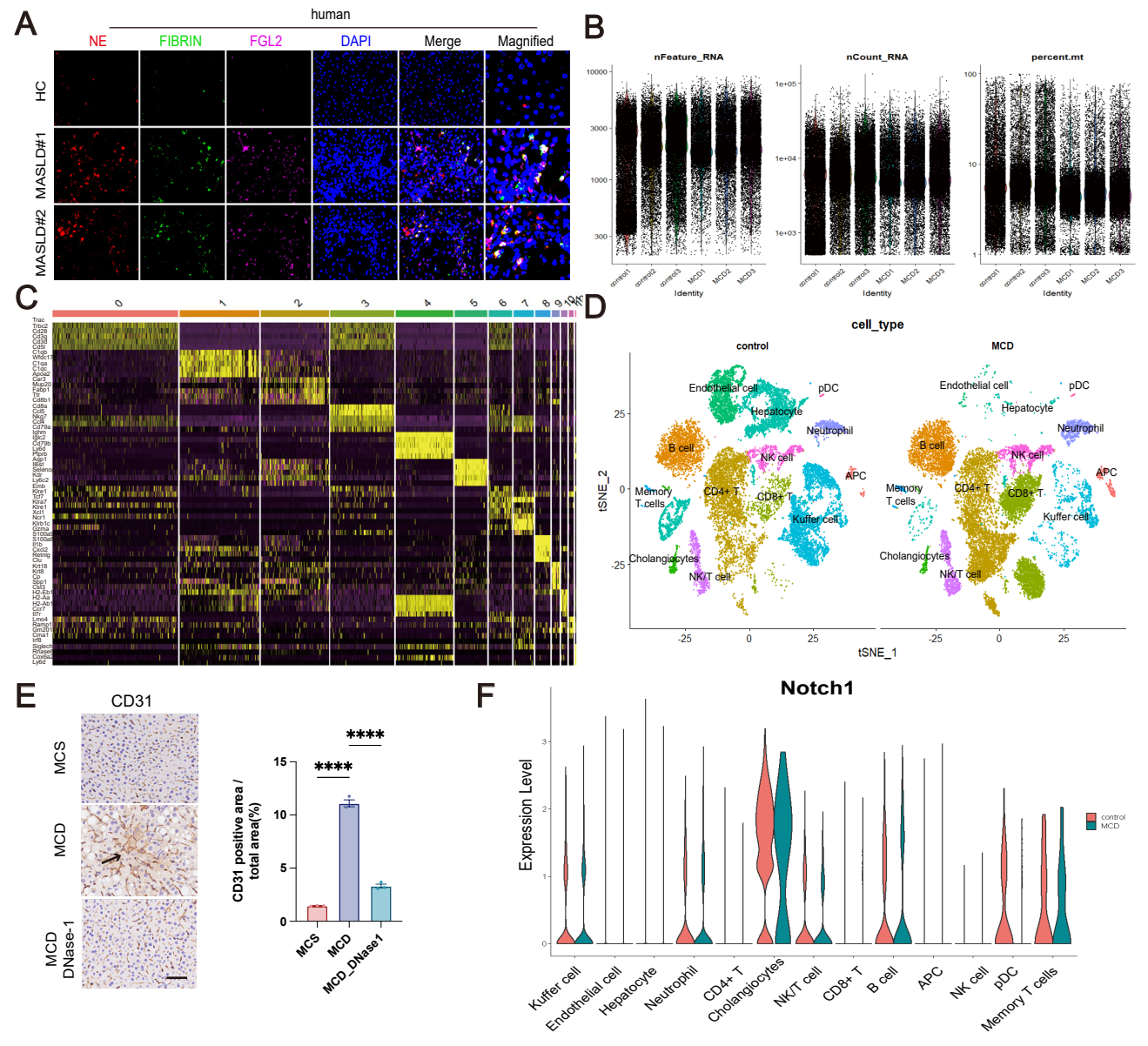

Supplement: Supplementary file 5 — Supporting File 5: advs75659‐sup‐0005‐Figure4.pdf. [file ADVS-9999-e22985-s006.pdf]

**A**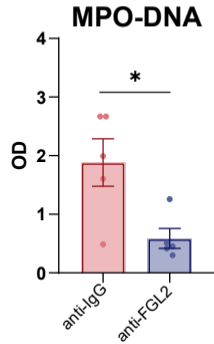**B**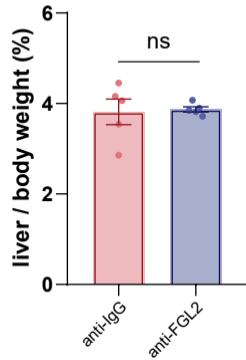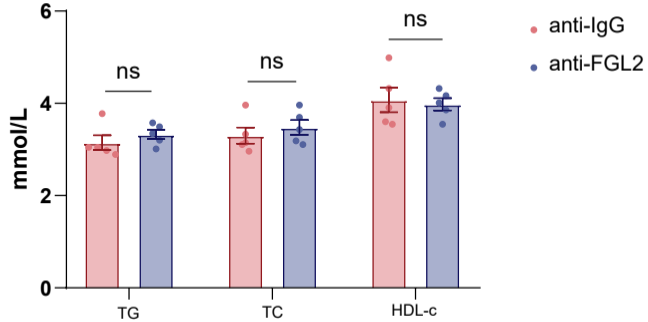

Supplement: Supplementary file 6 — Supporting File 6: advs75659‐sup‐0006‐Figure5.pdf. [file ADVS-9999-e22985-s003.pdf]
